# A model of anti-angiogenesis: differential transcriptosome profiling of microvascular endothelial cells from diffuse systemic sclerosis patients

**DOI:** 10.1186/ar2002

**Published:** 2006-07-19

**Authors:** Betti Giusti, Gabriella Fibbi, Francesca Margheri, Simona Serratì, Luciana Rossi, Filippo Poggi, Ilaria Lapini, Alberto Magi, Angela Del Rosso, Marina Cinelli, Serena Guiducci, Bashar Kahaleh, Laura Bazzichi, Stefano Bombardieri, Marco Matucci-Cerinic, Gian Franco Gensini, Mario Del Rosso, Rosanna Abbate

**Affiliations:** 1 Department of Medical and Surgical Critical Care – DENOTHE, University of Florence, Florence, Italy; 2 Department of Experimental Pathology and Oncology – DENOTHE, University of Florence, Florence, Italy; 3 Department of Internal Medicine, University of Florence, Florence, Italy; 4 Division of Rheumatology, Medical College of Ohio, Toledo, Ohio, USA; 5 Department of Internal Medicine, University of Pisa, Pisa, Italy; 6 Centro S Maria agli Ulivi, Fondazione Don Carlo Gnocchi, ONLUS IRCCS, Impruneta, Florence, Italy

## Abstract

The objective of this work was to identify genes involved in impaired angiogenesis by comparing the transcriptosomes of microvascular endothelial cells from normal subjects and patients affected by systemic sclerosis (SSc), as a unique human model disease characterized by insufficient angiogenesis. Total RNAs, prepared from skin endothelial cells of clinically healthy subjects and SSc patients affected by the diffuse form of the disease, were pooled, labeled with fluorochromes, and hybridized to 14,000 70 mer oligonucleotide microarrays. Genes were analyzed based on gene expression levels and categorized into different functional groups based on the description of the Gene Ontology (GO) consortium to identify statistically significant terms. Quantitative PCR was used to validate the array results. After data processing and application of the filtering criteria, the analyzable features numbered 6,724. About 3% of analyzable transcripts (199) were differentially expressed, 141 more abundantly and 58 less abundantly in SSc endothelial cells. Surprisingly, SSc endothelial cells over-express pro-angiogenic transcripts, but also show up-regulation of genes exerting a powerful negative control, and down-regulation of genes critical to cell migration and extracellular matrix-cytoskeleton coupling, all alterations that provide an impediment to correct angiogenesis. We also identified transcripts controlling haemostasis, inflammation, stimulus transduction, transcription, protein synthesis, and genome organization. An up-regulation of transcripts related to protein degradation and ubiquitination was observed in SSc endothelial cells. We have validated data on the main anti-angiogenesis-related genes by RT-PCR, western blotting, *in vitro *angiogenesis and immunohistochemistry. These observations indicate that microvascular endothelial cells of patients with SSc show abnormalities in a variety of genes that are able to account for defective angiogenesis.

## Introduction

Systemic sclerosis (SSc) affects the connective tissue of the skin and internal organs, such as gastrointestinal tract, lungs, heart and kidneys. Disease progression involves the immune system, extracellular matrix (ECM) deposition and the microvasculature [1]. In the later stages of the disease, the vessel walls are thickened and hyalinized and their lumen is narrowed, leading to devascularization and tissue ischemia, which is not counterbalanced by active neo-angiogenesis. Angiogenesis, the process of new blood vessel generation from capillary or post-capillary venules, requires gross changes in endothelial cell function. In this process, an endothelial cell modifies the interaction with its basement membrane, remodels and migrates through ECM, proliferates, and differentiates. The final effect is the formation of endothelial tubules with a lumen, which are capable of transporting blood [2]. Newly expressed molecules or hyper-expression of pre-existing ones are coordinately required in this series of events, including proteolytic enzymes that are believed to be critical to ECM remodeling [3], growth factor activation [4] and release of ECM-trapped regulatory molecules [5,6].

While gene-expression profiling using microarray technologies is available for skin biopsies [7] and cultured fibroblasts from individuals with a diagnosis of SSc [8,9], a global portrait of gene expression of microvascular endothelial cells (MVECs) has not been reported in the literature. In order to better understand whether dysregulated genes may contribute to the pathogenesis of defective angiogenesis, we have undertaken studies of gene expression in MVECs isolated from the lesional skin of patients affected by the diffuse form of SSc and matched healthy controls, using a 14,000 oligonucleotide (70 mer) microarray. After the identification of differentially expressed genes by a Bayesian empirical model [10,11], genes were annotated on the basis of biological process ontology and statistically significant gene ontology terms were evaluated.

The results show that of the several thousands genes that passed filtering criteria, 199 genes are differentially expressed, 141 being up-regulated and 58 down-regulated in SSc endothelial cells. We observed that SSc endothelial cells overexpress pro-angiogenic and anti-angiogenic transcripts, and down-regulation of genes critical to cell migration and proliferation (including tissue kallikreins (KLKs)) [12], adhesion and capillary differentiation. We have validated the data on the main anti-angiogenesis-related molecules by RT-PCR and have focused functional experiments on differentially expressed molecules that have recently been shown to be relevant to endothelial cell physiology, such as plexin B1, pent(r)axin 3 and desmoglein (DSG) 2. Plexin B1, which we found to be down-regulated in MVECs of SSc patients, has been reported to bind and mediate the pro-angiogenic signal of semaphorin 4D [13]. Pent(r)axin 3, which we found to be up-regulated in MVECs of SSc patients, inhibits the pro-angiogenic effect of Fibroblast Growth Factor-2 (FGF2), including that produced by autocriny of endothelial cells [14]. Desmoglein 2 is a calcium-binding trans-membrane protein of the cadherin cell adhesion molecule superfamily that mediates homophilic cell adhesion, and has been identified as a structural component of endothelial cell intercellular junctions [15]. Here we show that DSG2 down-regulation in MVECs of SSc subjects associates with an anti-angiogenic phenotype.

We also identified differential expression of transcripts controlling haemostasis, inflammation, stimulus transduction, transcription, protein synthesis and genome organization. Other up-regulated genes are markers of cellular stress, such as those of the ubiquitin-proteasoma family. Taken together, our results show that over-expression of some genes in SSc MVECs indicates a response to a powerful pro-angiogenic environment, while over-expression of others may render them unable to respond to angiogenic stimuli by over-expression of anti-angiogenic and hypo-expression of pro-angiogenic molecules.

## Materials and methods

### Patients, controls, tissue biopsies and endothelial cell preparation

Patients with diffuse SSc (submitted to skin biopsies for diagnostic purposes at the Departments of Medicine, Division of Rheumatology, Florence and Pisa Universities) and healthy controls were used as sources of MVECs. All patients (20 females and 10 males, with a mean disease duration of 9 years (range 3.1 to 12)) fulfilled the American College of Rheumatology criteria for the classification of SSc [16]. Only patients classified as having the diffuse cutaneous SSc were admitted to the study (sclerosis of both distal and proximal extremities, with or without truncal involvement). Patients with overlap symptoms to other connective diseases were excluded from the study, as well as patients affected by other diseases involving the vascular system. Biopsies were performed on the dorsal involved skin of the hands. Fifteen healthy patients undergoing surgery for traumatic events involving the hands were subjected to the MVEC isolation procedure, after punch biopsies of the dorsal skin of the hands, which were processed as skin biopsies of SSc patients. The study was approved by the local Ethical Committee and patient consent was obtained from each subject enrolled. Ethics approval and patient consent were granted for this manuscript.

The patients were not on steroids, cyclophosphamide, D-penicillamine, relaxin or other disease-modifying drugs. Calcium channel blockers were stopped ten days before the biopsy. Only proton pump inhibitors and cisapride were allowed. Briefly, skin biopsies have been mechanically cleaned of epidermis and adipose tissue in order to obtain a pure specimen of vascularized dermis, and treated as described elsewhere [17,18]. In some cases, clusters of round-shaped cells were squeezed from microvessels and formed colonies composed by polygonal elements. Such colonies were detached with EDTA, and CD31-positive cells were subjected to immuno-magnetic isolation with Dynabeads CD31 (Dynal ASA, Oslo, Norway) [18]. Isolated cells were further identified as MVECs by labeling with anti-factor VIII-related antigen and by re-probing with anti-CD31 antibodies. Cells were maintained in complete MCDB medium, supplemented with 30% FCS, 20 μg/ml endothelial cell growth supplement (ECGS), 10 μg/ml hydrocortisone, 15 UI/ml heparin, and antibiotics (100 UI/ml penicillin, 100 μg/ml streptomycin, 50 μg/ml amphotericin). MVECs from normal individuals and from SSc patients are referred to as N-MVECs and SSc-MVECs, respectively, and were used between the third and seventh passage in culture. A biopsy specimen from each subject was formalin fixed and paraffin embedded for immunohistochemistry assays. Each case has been stained with hematoxilin and eosin to assess the original diagnosis.

### RNA preparation

Since the success rate for isolation of SSc-MVECs is lower than 20%, compared to a success rate of more than 50% for N-MVEC, controls were matched by age and sex to the SSc cases that yielded MVECs. Therefore, total RNA was prepared from six N-MVEC and six SSc-MVEC pellets using the RNeasy Minikit (Qiagen, Hilden, Germany) according to the manufacturer's protocol. Equal quantities of total RNA from each of the six N-MVEC and six SSc-MVEC pellets were pooled to give a N-MVEC pool and a SSc-MVEC pool.

### Microarray based gene expression analysis

The setting and the subsequent hybridization of microarrays were performed as described in a previous paper [12]. Briefly, we used poly-L-lysine (Sigma Chemical Company, St. Louis, MO, USA) coated arrays representing 14,000 genes (70 mer oligonucleotides; Human Array-Ready Oligo set version 1.1, Operon Technologies, Inc., Alameda, CA, USA). We reverse transcribed and labeled 20 μg of the N-MVEC pool and 20 μg of the SSc-MVEC pool with NHS-cyanine dyes (Cy3 and Cy5; Amersham Biosciences, Amersham Place, England). The two labeled probes were hybridized with the array for 16 h at 65°C. Arrays were scanned by using a 4000B Scanner (Axon Instruments, Union City, CA, USA). Due to difficulty in growing SSc-MVECs, we performed two replicates of the microarray experiment: one with N-MVECs Cy3 labeled and SSc-MVECs Cy5 labeled and one with N-MVECs Cy5 labeled and SSc-MVECs Cy3 labeled (dye swap).

### Image processing and statistical analysis

Each hybridization produced a pair of 16-bit images, which were processed using the GenePix Pro 4.1 software (Axon Instruments). Poorly spotted genes, expressing weak or distorted signals, were automatically discarded by the GenePix Pro 4.1 software and manually by visual inspection. In order to reduce the identification of false positive differentially expressed genes, spots exhibiting at the same time low Cy3 and Cy5 fluorescent signal intensities (<100) were discarded from consideration by pre-processing the raw data in Axon .gpr format using Microsoft Excel. For each microarray, we performed a local intensity-dependent normalization using an lowess scatter plot smoother to remove dye and spatial (print-tip) effects [19]. After single-slide normalization we applied a dye-swap normalization as proposed by Kerr and colleagues [11,20] to correct the different properties of the dyes on a gene by gene basis [21]. The data obtained from normalization was analyzed by Newton algorithm [10]. More details on image processing and statistical analysis have been previously reported [12]. The full data set is available at ArrayExpress [22].

### Gene ontology data analysis

In our study we used one of the three ontologies produced by the Gene Ontology (GO) consortium, the biological process ontology. The term 'biological process' should be interpreted as a biological function to which the gene product contributes. The actual mappings of genes to GO terms are provided by the Gene Ontology Annotation Database [23,24]. The mappings were downloaded from [25].

In brief, given a set of genes and one ontology, we first found the set of all unique GO terms within the ontology that are associated with one or more of the genes of interest. Next, we determined how many of the selected 199 differentially expressed genes are annotated at each term and how many of the genes that were assayed (all the genes represented on the microarray) are annotated at the term. The test evaluated if there are more interesting genes at the term than one might expect by chance. Due to the small number of genes in some categories, significant terms were inferred by two-sided Fisher's exact test [26]. The statistical analyses were implemented in the R environment using Bioconductor packages [27].

Criteria based only on GO terms were not sufficient to classify a gene as positively or negatively involved in the regulation of angiogenesis. Therefore, we included the biological processes obtained by GO into the following families: angiogenesis, apoptosis, haemostasis, inflammation and immunity, stress and ubiquitination, transductions, DNA/RNA organization, transcription, protein synthesis, and mitochondrial functions. In particular, in order to be classified as pro-angiogenic, a gene must play a significant role in endothelial cell adhesion, invasion, proliferation, and differentiation.

### Real Time RT-PCR based gene expression analysis

In order to confirm results obtained by microarray analysis, the expression patterns of nine selected genes were also measured by reverse transcription (RT)-PCR. For RT-PCR, 7 μg of the total RNA pools used for comparative microarray experiments were reverse-transcribed using M-MLV transcriptase (Gibco BRL, Gaithersburg, MD, USA) and random hexamer primers (Amersham). To quantify the transcribed IL8, PLAU, KLK9, KLK11, KLK12, PTX3, PLXNB1, DSG2, and CTGF genes, we performed TaqMan RT-PCR (Applied Biosystems, Foster City, CA, USA) on an ABI Prism 7700 instrument. VIC-labeled human glyceraldehyde-3-phosphate dehydrogenase (GAPDH; #4326317E) and FAM-labeled human IL8 (#Hs00174103_m1), urokinase type plasminogen activator (PLAU; #Hs00705898_s1) KLK9 (#Hs00705898_s1), KLK11 (#Hs00170182_m1 and Hs00374668_m1), KLK12 (#Hs00377603_m1), PTX3 (#Hs00173615_m1), PLXNB1 (#Hs00182227_m1), DSG2 (#Hs00170071_m1), and connective tissue growth factor (CTGF; #Hs00170014_m1) TaqMan pre-developed assays (Applied Biosystems, Foster City, CA, USA) were used. Expression of IL8, PLAU, KLKs, PTX3, PLXNB1, DSG2, and CTGF genes was normalized to GAPDH and displayed as fold-change relative to N-MVEC RNA used as the calibrator. Reactions were performed in duplicate with 200 ng cDNA. The experiment was repeated in two independent runs. ΔCt values of the samples were determined by subtracting the average of the duplicate Ct values of the target genes from the average of the duplicate Ct values of the GAPDH gene (reference). The relative gene expression levels were determined by subtracting the average ΔCt value of the target from the average ΔCt value of the calibrator. The amount of target (expressed as fold change), normalized to an endogenous reference and relative to a calibrator, was given by 2^-ΔΔCt^. Moreover, for all the genes reported in Table 1, 7 μg of total RNA from MVECs from the six individual SSc patients and the six healthy subjects was reverse-transcribed and analyzed.

### Immunohistochemistry

For immunohistochemistry, tissue sections were 3 to 5 μm thick and placed on pretreated glass slides, dewaxed and treated to block endogenous peroxidase activity. The following primary antibodies were employed: rabbit anti-human KLK9 (catalog n K005-12, raised against a synthetic peptide corresponding to amino acids 239 to 250 of the human KLK9 protein) and anti-human KLK12 (catalog n K005-15, raised against a synthetic peptide corresponding to amino acids 236–248 of the human KLK12 protein), and mouse anti-human KLK11 (catalog n K005-14, raised against human recombinant KLK11), all from US Biological (Swampscott, MA, USA); anti-DSG mouse monoclonal antibody (Chemicon, Temecula, CA, USA); anti-pentraxin 3 rat monoclonal antibody MNB4 (Alexis Biochemical, Lausen, Switzerland); anti-plexin B1 and anti-CTGF rabbit polyclonal antibodies, both from Santa Cruz Biotechnology (Santa Cruz, CA, USA); murine monoclonal antibody 5B4 (mAb5B4), which recognizes the kringle domain of the A chain of PLAU, a kind gift of Dr ML Nolli (Lepetit Research Center, Varese, Italy); and anti-IL8 rabbit polyclonal antibodies (Chemicon, Temecula, CA, USA). All the primary antibodies were diluted 1:40 and incubated overnight with tissue sections in a moist chamber at 4°C. A standard streptavidin-biotin detection system (Vector, Burlingame, CA, USA) was carried out. Isotype Ig controls were used in parallel with primary antibodies to assess the specificity of the staining. Primary antibody bound to antigen was visualized by diaminobenzidine staining and a nuclear counterstaining with hematoxilin was performed. Immunohistochemistry was performed on the skin biopsies of the six normal subjects and six SSc patients whose MVECs were used for RNA preparation. Immunohistochemistry quantification was performed by image analysis using the ScnImage program [28].

### Western blotting

N-MVECs and SSc-MVECs were grown to 70% confluence and serum-starved overnight in MCDB supplemented with 2% FCS. Cells were then suspended in lysis buffer (10 mM Tris-HCl, pH 7.4, containing 150 mM NaCl, 1% Triton X-100, 15% glycerol, 1 mM sodium orthovanadate, 1 mM NaF, 1 mM EDTA, 1 mM phenylmethylsulfonyl fluoride, and 10 μg/ml aprotinin). We electrophoresed 60 μg of the cell extract proteins on 12% SDS-PAGE under reducing conditions and then blotted to a polyvinylidene difluoride membrane (Hybond-C Extra; Amersham Biosciences, Piscataway, NJ, USA) for 3 h at 35 V. The membrane was incubated with 5% skim milk in 20 mM Tris buffer, pH 7.4, for 1 h at room temperature to block non-specific binding and then probed with antibodies directed against pentraxin 3, or plexin B1 or DSG2 overnight at 4°C. After incubation with horseradish peroxidase-conjugated donkey anti-mouse IgG (1:5,000) for 1 h (Amersham Biosciences), immune complexes were detected with the enhanced chemiluminescence detection system (Amersham Biosciences). The membranes were exposed to autoradiographic films (Hyperfilm MP; Amersham Biosciences) for 1 to 30 minutes.

### Migration assays

A Boyden chamber was used to evaluate spontaneous and stimulated invasion (chemoinvasion) through Matrigel-coated porous filters, as described [12]. For spontaneous invasion, 50 μl of cell suspension (6.25 × 10^3 ^cells) were placed in the upper compartment of the chamber and migration was allowed to occur for 6 h. To inhibit the activity of the relevant molecules, specific antibodies (anti-pentraxin 3 and anti-plexin B1, each at 3 μg/ml final concentration) were added to both the upper and lower compartment of the migration chamber. Irrelevant mouse IgGs were used to verify the specificity of the effect. The number of cells moving across the filter measured mobilization. Experiments were performed in triplicate. Migration was expressed as mean ± standard deviation (SD) of the number of total cells counted per filter or as the percentage of basal response.

### Preparation of SSc-MVEC conditioned medium

Confluent cultures of SSc-MVECs were washed twice with phosphate-buffered saline and incubated overnight in the presence of MCDB medium supplemented with 2% FCS. The culture supernatant was centrifuged at 1,500 rpm for 10 minutes and either used immediately or stored at -20°C.

### *In vitro *capillary morphogenesis assay

Matrigel (0.5 ml; 10 to 12 mg/ml) was pipetted into 13 mm diameter tissue culture wells and polymerized for 30 minutes to 1 h at 37°C, as described [12]. N-MVECs were plated (60 × 10^3^/ml) in complete MCDB medium supplemented with 30% FCS and 20 μg/ml endothelial cell growth supplement. Capillary morphogenesis was also performed in the presence of 3 μg/ml of anti-pentraxin 3 or anti-plexin B1 antibody. Irrelevant mouse IgGs were used as negative control. Plates were photographed at 24 h. Results were quantified by measuring the percentage of the photographic field occupied by endothelial cells by image analysis. Six to nine photographic fields from three plates were scanned for each point.

### Statistical analysis

Results are expressed as means ± SD for (n) experiments. Multiple comparisons were performed by the Student-Newman-Keuls test, after demonstration of significant differences among medians by nonparametric variance analysis according to Kruskal-Wallis.

## Results

### Microarray, gene ontology analysis, and class distribution of differentially expressed genes

Of the 14,000 transcripts represented on our arrays, after data processing and application of the filtering criteria, the analyzable features numbered 6,724. The full list of the 150 most expressed genes, independent of the cellular source (N-MVEC and SSc-MVEC), is available as Additional file 1a. We used a Newton algorithm after single slide and dye-swap normalization to assess the 6,724 genes expressed by MVECs for differential expression between SSc-MVECs and N-MVECs.

Genes found differentially expressed between SSc-MVECs and N-MVECs numbered 199 (3% of the total transcripts analyzed; Additional files 2 to 7). Of these, 141 transcripts were expressed more abundantly and 58 less abundantly in the SSc-MVECs.

To analyze the involvement of differentially expressed genes in different biological functional groups, all the genes present on the microarray were annotated for their biological processes. According to GO analysis, we observed 55 significant terms (*P *value <0.05) associated with genes differentially expressed in SSc (Table 2). In Table 2, significant terms with more than two annotated genes (N) on the array are reported; also, for each significant GO term, the symbols of the genes are reported. The full list of GO terms for all the differentially expressed genes is available in Additional file 1b.

The GO biological processes include many of those known to be required to fulfill an angiogenic program. Of particular interest are the genes involved in proteolysis and peptidolysis, cell migration and cell motility, Rho protein signal transduction, regulation of cell adhesion, blood coagulation, and mitosis.

However, many of the differentially expressed genes have multiple functions, each one often required for angiogenesis, and some recognized pro- or anti-angiogenic properties of several genes are not yet available in the GO biological processes.

Because of this, we decided to further classify the differentially expressed genes according to a series of criteria that take into consideration a recognized role of the relevant encoded protein in the biological processes shown in Table 3: angiogenesis (Table 3 and Additional file 2), including cell invasion, proliferation, adhesion, differentiation, and inhibition of angiogenesis; apoptosis, haemostasis, inflammation and immunity (Table 3 and Additional file 3); cellular stress and ubiquitination (Table 3 and Additional file 4); transductions, DNA/RNA organization, and regulation of transcription (Table 3 and Additional file 5); and regulation of protein synthesis and mitochondrial functions (Additional file 6). Each gene endowed with multiple functions is mentioned in more than a single additional file.

Table 3, which reports differentially expressed genes with a log odds ratio (LOR) >1.0 (see also Additional file 2), where gene function was classified according to GO and to information available in the NCBI web site and related links [29,30], indicates that many genes that mediate endothelial cell migration/invasion, proliferation, cytoskeletal remodeling and capillary differentiation (angiogenesis section) are up-regulated in SSc-MVECs. However, the angiogenesis inhibitor pent(r)axin-related gene (*PTX3*) is also up-regulated, while other genes critical for the angiogenic process, such as plexin B1 (*PLXNB1*, semaphorin receptor), tissue kallikreins KLK9, KLK11, and KLK12) [12], and DSG2 (a cadherin that mediates homophilic cell adhesion), undergo down-regulation in SSc-MVECs. Apoptosis-related genes (Table 3 and Additional file 3) were variously altered, including down-regulation of *BCL2 *in SSc-MVECs, which also exhibited a general pro-fibrinolytic pattern, coupled with over-expression of *PTX3*, which increases tissue factor expression and stimulates generation of inflammation mediators, [31]. SSc-MVECs also show up-regulation of genes related to a response to oxidative, osmotic, and shear stress, and of genes linked with protein ubiquitination and proteasoma activation (Table 3 and Additional file 4). Table 3 and Additional file 5 indicate an overall perturbation of signal transductions mediated by small GTPase proteins, and down-regulation of MAP4K in SSc-MVECs, and of genes involved in nucleosome and chromatin remodeling and in regulation of transcription, including down-regulation of GATA6, which controls transcription of von Willebrand factor, and of RUNX2, which controls endothelial cell migration and invasion. Table 3 and additional file 6 show up-regulation in SSc-MVECs of a large number of structural constituents of ribosomes and of genes engaged in oxidative phosphorylation and related ATP production, indicating an intense protein synthesis and energy production in SSc-MVECs. A series of 36 differentially expressed genes whose functions are unknown or cannot be included within a class is available as Additional file 7.

### Validated expression of selected genes by RT-PCR

To validate the results of the cDNA microarray analysis, the mRNA expression of nine selected genes was independently examined with real time RT-PCR. We selected nine differentially expressed transcripts, including many of those that are functional to the main hypothesis of the present work (KLK9, AF135026; KLK11, AB012917; KLK12, AF135025; IL8, M17017; PLAU, X02419; PTX3, M31166; PLXNB1, AJ011414; DSG2, NM_001943; CTGF, NM_001901); among these were genes exhibiting a significant decrease (KLK9, KLK11, KLK12, PLXNB1, and DSG2) or a significant increase (IL8, PLAU, PTX3, and CTGF) in expression in SSc-MVECs in comparison to N-MVECs. We evaluated these in the same total RNAs used for comparative microarray experiments. Real time RT-PCR analysis confirmed the data obtained by microarray technology (Table 1). To reinforce the data on the genes reported in Table 1, we also performed RT-PCR determinations on single RNA preparations (from six SSc patients and six healthy subjects), as previously described for single KLKs [12]. The values obtained from these determinations were similar to those obtained from the RNA pools: a mean fold increase for PTX3 (1.72, range 1.18 to 4.73, p = 0.041), IL8 (3.3, range 1.51 to 7.9, p = 0.039), PLAU (2.76, range 1.51 to 5.2, p = 0.02), and CTGF (1.79, range 1.19 to 3.09, p = 0.026); and a mean fold decrease for PLXNB1 (1.96, range 1.35 to 5.91, p = 0.042), DSG2 (29.91, range 10.62 to 68.9, p = 0.02), KLK9 (20.69, range 3.82 to 75.0, p = 0.022), KLK11 (34.48, range 5.83 to 150.0, p = 0.021), and KLK12 (24.26, range 2.64 to 118.0, p = 0.020).

### Immunohistochemistry

**Figure 1:**
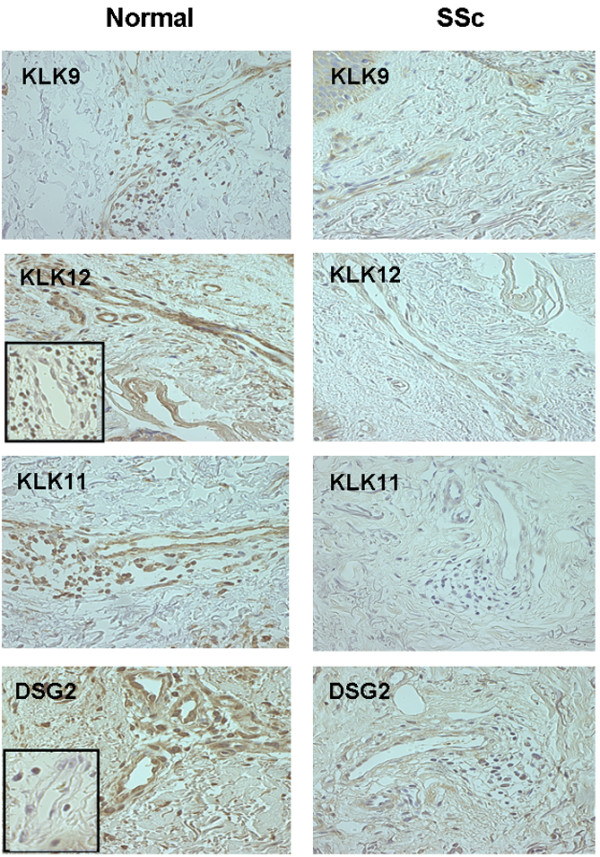
Immunohistochemical evidence of differential expression of tissue kallikrein (KLK)9, KLK11, KLK12, and DSG2 in microvascular endothelial cells (MVECs) from normal subjects and patients with systemic sclerosis (SSc). Each panel is representative of the pattern of immune staining with each antibody obtained for the six normal and six SSc patients subjected to skin biopsies from which MVECs were prepared. The inset in the micrograph of KLK12 shows negative staining using isotype control rabbit IgG (the isotype used for KLK9 and KLK12), while that shown in the micrograph of DSG shows negative staining for mouse IgG (the isotype used for KLK11 and DSG). Original magnification, 200×.

On the basis of RT-PCR differential expression of the relevant genes, we performed an immunohistochemistry analysis of the nine validated molecules. In spite of the scarcity of microvessels in the lesional skin biopsies of SSc patients, all tissue samples from both normal (six biopsies) and SSc (six biopsies) subjects showed the presence of endothelial cells exhibiting immunoreactivity for KLK9, KLK11, KLK12, DSG2, plexin B1, IL8, PLAU, pent(r)axin 3, and connective tissue growth factor. The sensitivity of the method did not enable us to identify a differential immuno-staining for molecules whose RT-PCR expression showed differences ranging from 42% (CTGF up-regulation in SSc-MVECs, Table 1) to 185% (IL8 up-regulation in SSc-MVECs, Table 1), while all the tissue KLKs and DSG2, whose down-regulation in SSc-MVECs ranged from 10.63-fold to 53.07-fold, exhibited a measurable differential staining (Figure 1). Due to the poor presence of microvessels in SSc biopsies, an average of three vessels per biopsy was subjected to image analysis. Evaluation of differential staining intensity gave the following results: KLK9, 47.2 ± 15% decrease in SSc (p < 0.05); KLK11, 69.7 ± 21% decrease in SSc (p < 0.05); KLK12, 61.6 ± 23% decrease in SSc (p < 0.05); DSG2, 62.7 ± 14% decrease in SSc (p < 0.05). Isotype controls stained negative, as shown in the insets of Figure 1 (rabbit Ig G for KLK9 and KLK12, and mouse IgG for KLK9 and DSG).

### Functional studies on the angiogenic effects of pentraxin 3, plexin B1 and DSG2

**Figure 2:**
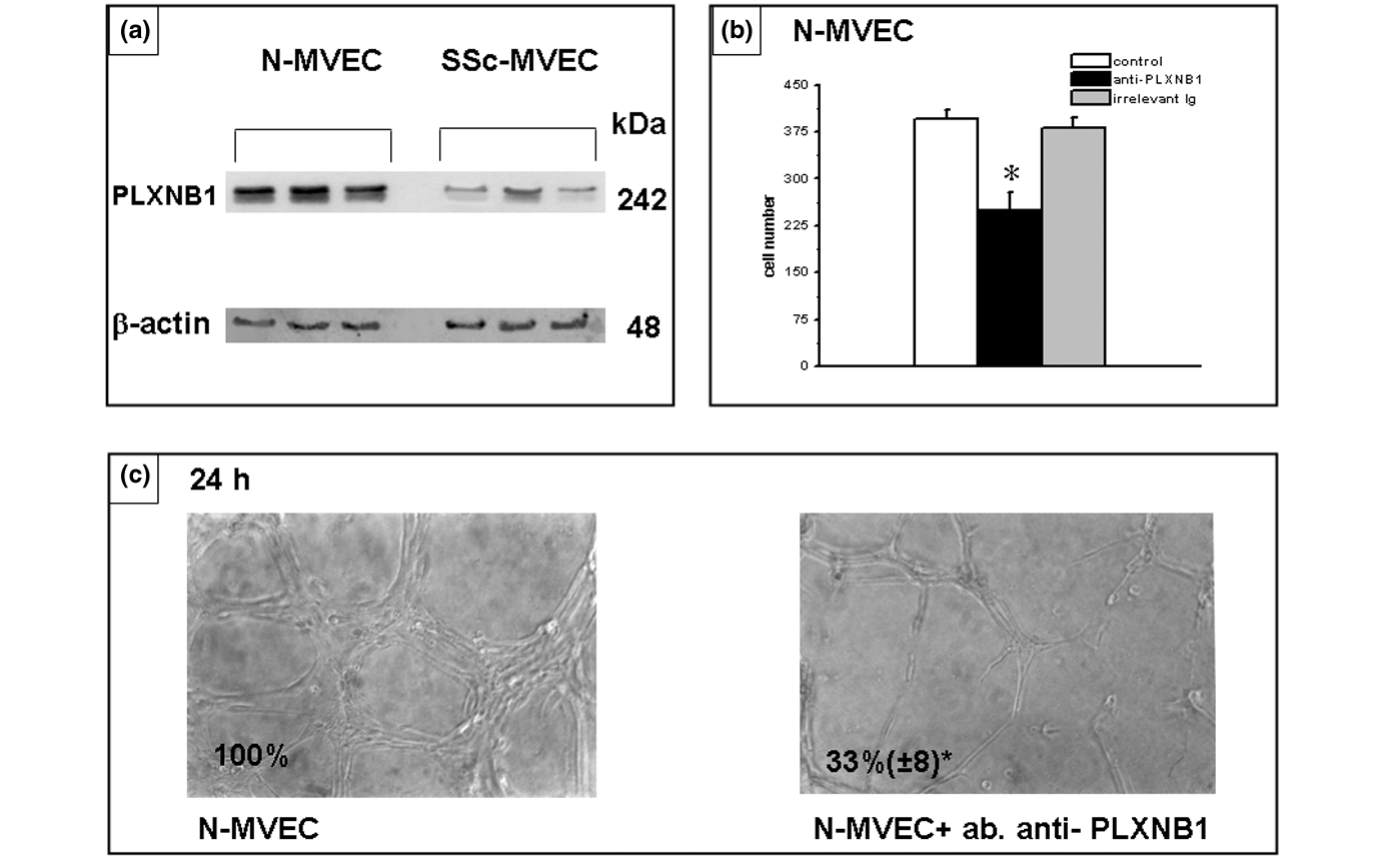
Expression and role of plexin B1 (PLXNB1) in endothelial cell invasion and capillary morphogenesis. (**a**) Western blotting of 60 μg protein from cell lysates of microvascular endothelial cells (MVECs) from normal subjects (N-MVECs) and patients with systemic sclerosis (SSc-MVECs) with anti-PLXNB1 antibodies. Each lane represents western blotting of MVECs obtained from a single patient. Actin was used as an internal reference standard. Numbers on the right represent the molecular weight expressed in kDa. (**b**) Effect of anti-PLXNB1 antibodies (3 μg/ml) on matrigel invasion of N-MVECs. The effect of irrelevant rabbit IgG is also shown. Numbers on the x-axis refer to the total number of cells migrated through the matrigel after 6 hours. Data are the mean ± standard deviation of three experiments performed in triplicate in three N-MVEC lines. The asterisk indicates that values are significantly different from the values of control (p < 0.05). (**c**) Effect of anti-PLXNB1 antibodies on capillary morphogenesis of N-MVECs. N-MVECs were plated on Matrigel (60 × 10^3^/ml), in complete MCDB medium, supplemented with 30% fetal calf serum, and 20 μg/ml endothelial cells growth supplement. N-MVEC spontaneously form anastomosing cords of cells resembling a capillary plexus, which are well organized by 6 hours. The process of endothelial cell organization after 6 hours is impaired in the presence of 3 μg/ml of anti-PLXNB1 rabbit polyclonal antibodies. Irrelevant rabbit IgG gave results similar to control untreated N-MVECs (not shown). These data are representative of three different experiments performed on three N-MVEC cell lines (100× magnification). Numbers reported within each panel indicate the percent of the photographic field occupied by cells ± standard deviation. The asterisk indicates that values are significantly different from the values of control N-MVECs at 6 hours after plating on Matrigel (p < 0.05).

**Figure 3:**
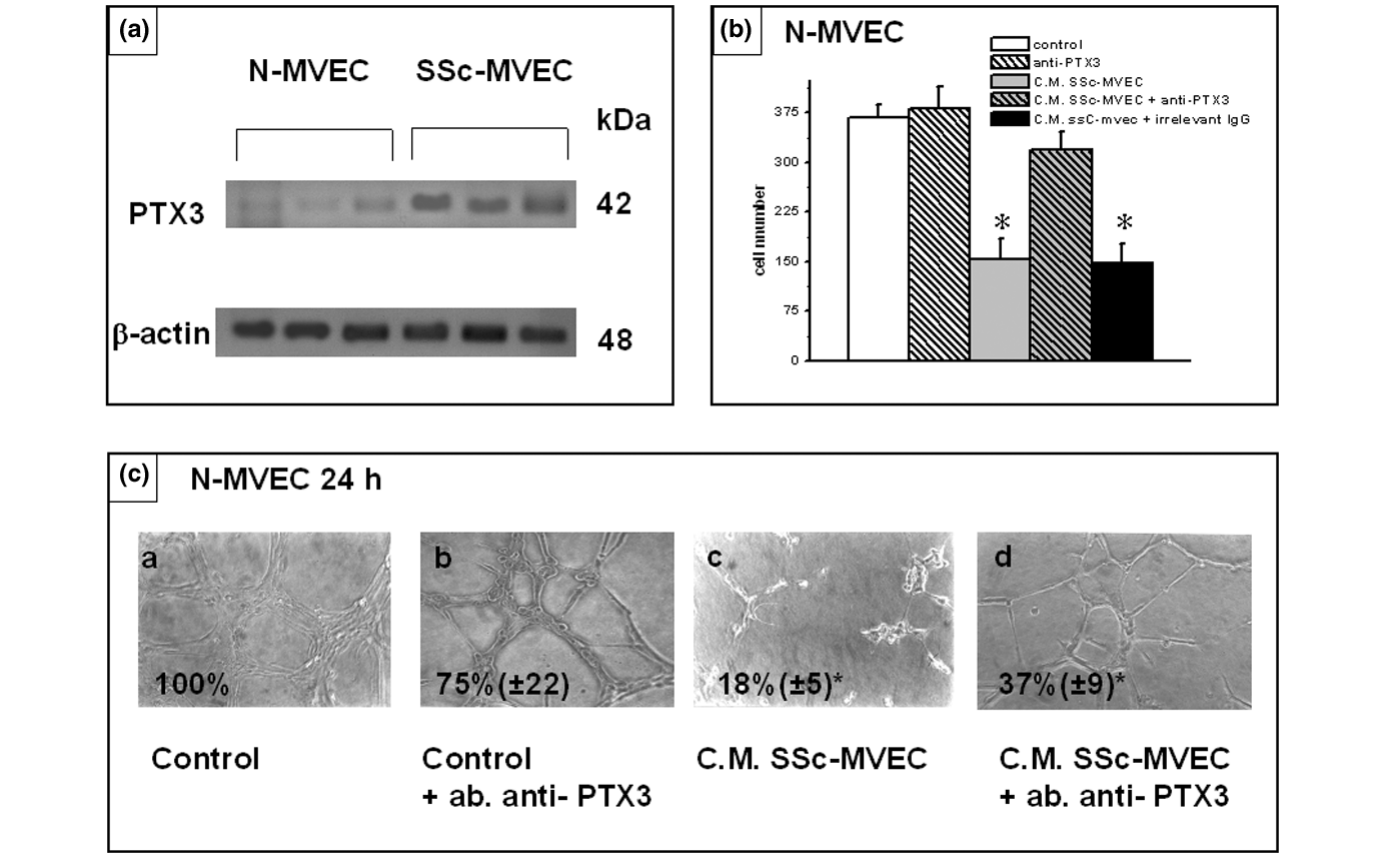
Expression and role of pent(r)axin 3 (PTX3) in endothelial cell invasion and capillary morphogenesis. (**a**) Western blotting of 60 μg protein from cell lysates of microvascular endothelial cells (MVECs) from normal subjects (N-MVECs) and patients with systemic sclerosis (SSc-MVECs) with anti-PTX3 antibodies. Each lane represents western blotting of MVECs obtained from a single patient. Actin was used as an internal reference standard. Numbers on the right represent the molecular weight expressed in kDa. (**b**) Effect of PTX3 antibodies (3 μg/ml) on matrigel invasion of N-MVECs. The effect of irrelevant rat IgG is also shown. Numbers on the x-axis refer to the total number of cells migrated through the matrigel after 6 hours. The presence of anti-PTX3 antibodies is able to revert inhibition of matrigel invasion induced by SSc-MVECs conditioned medium (C.M.). Data are the mean ± standard deviation of three experiments performed in triplicate in three N-MVEC lines. An asterisk indicates that values are significantly different from the values of control (p < 0.05). (**c**) Effect of anti-PTX3 antibodies on capillary morphogenesis of N-MVECs. Experimental conditions were as described in the legend to Figure 1. Conditioned medium from SSc-MVECs was able to impair capillary morphogenesis observed after 6 hours from plating (panel c). The process of endothelial cell organization after 6 hours was partially restored in the presence of 3 μg/ml of anti-PTX3 rat monoclonal antibodies (panel d), which showed only a small effect when added to control N-MVECs (panel b). Irrelevant rat IgG did not show any effect (not shown). These data are representative of three different experiments performed on three N-MVEC cell lines (100× magnification). Numbers reported within each panel indicate the percent of the photographic field occupied by cells ± standard deviation. An asterisk indicates that values are significantly different from the values of control N-MVECs at 6 hours after plating on Matrigel (p < 0.05).

**Figure 4:**
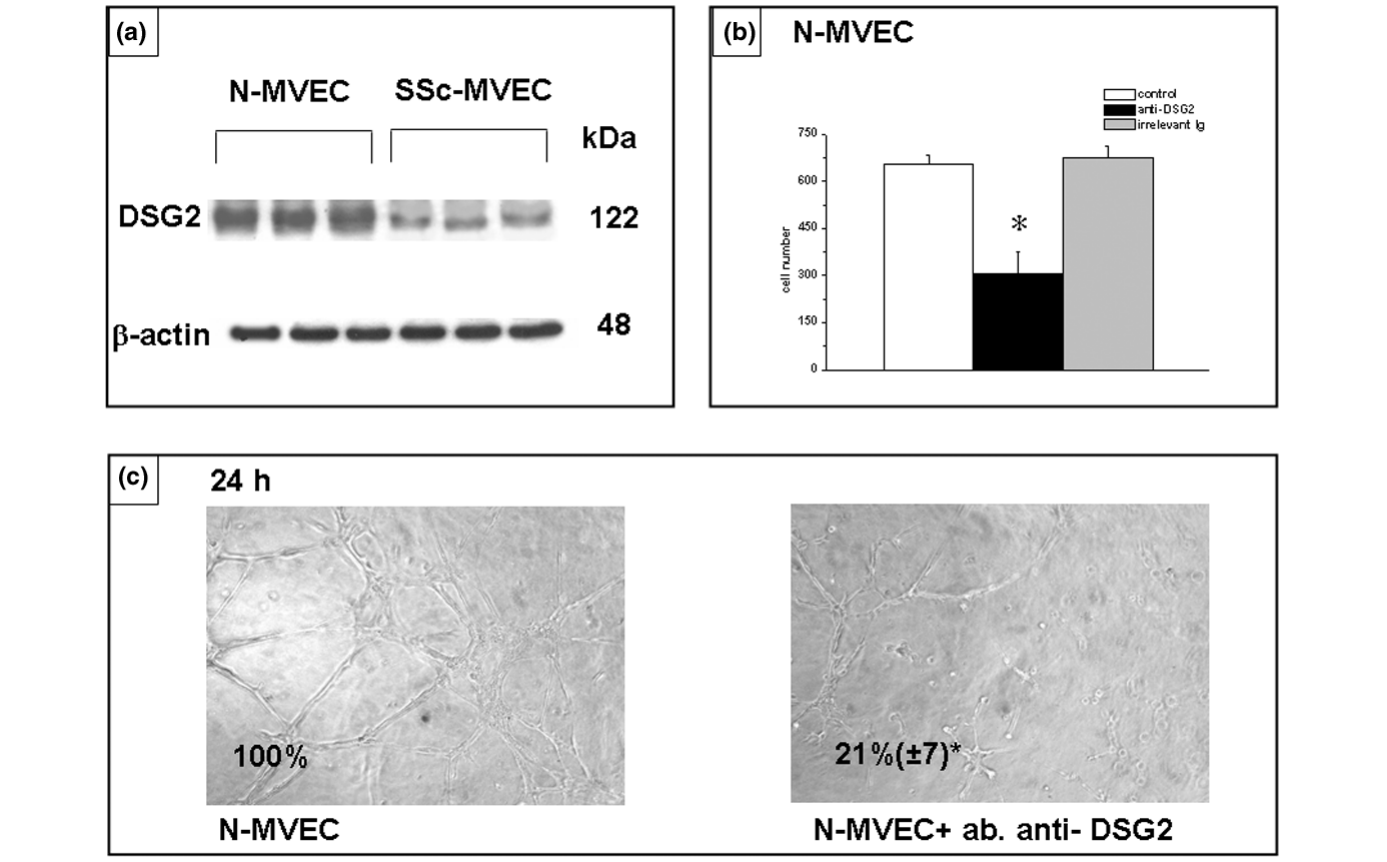
Expression and role of desmoglein (DSG)2 in endothelial cell invasion and capillary morphogenesis. (**a**) Western blotting of 60 μg protein from cell lysates of microvascular endothelial cells (MVECs) from normal subjects (N-MVECs) and patients with systemic sclerosis (SSc-MVECs) with anti-DSG2 antibodies. Each lane represents western blotting of MVECs obtained from a single patient. Actin was used as an internal reference standard. Numbers on the right represent the molecular weight expressed in kDa. (**b**) Effect of anti-DSG2 antibodies (3 μg/ml) on matrigel invasion of N-MVECs. The effect of irrelevant mouse IgG is also shown. Numbers on the x-axis refer to the total number of cells migrated through the matrigel after 6 hours. Data are the mean ± standard deviation of three experiments performed in triplicate in three N-MVEC lines. The asterisk indicates that values are significantly different from the values of control (p < 0.05). (**c**) Effect of anti-DSG2 antibodies on capillary morphogenesis of N-MVECs. See the legend to Figure 1 for experimental details. Irrelevant mouse IgG gave results similar to control untreated N-MVECs (not shown). These data are representative of three different experiments performed on three N-MVEC cell lines (100× magnification). Numbers reported within each panel indicate the percent of the photographic field occupied by cells ± standard deviation. The asterisk indicates that values are significantly different from the values of control N-MVECs at 6 hours after plating on Matrigel (p < 0.05).

We have previously shown down-regulation at the protein level of tissue KLK9, KLK11 and KLK12, as well as how such alterations account for reduced angiogenesis in SSc-MVECs [12]. Here we have focused our studies on the role of pent(r)axin 3, plexin B1, and DSG2, three gene products that are particularly relevant to the hypothesis of the present study. Although the 90% decrease of PLXB1 and 58% increase of PTX3 mRNA in SSc-MVECs (Table 1) were not demonstrable by differential immuno-staining of endothelial cells in tissue biopsies, the differential protein expression and their functional import were evident by western blotting and *in vitro *angiogenesis assays. Figures 2a, 3a and 4a, which show western blotting of cell lysates with anti-plexin B1, anti-pent(r)axin 3 and anti-DSG2 antibodies, respectively, indicate down-regulation of plexin B1 and DSG2, and up-regulation of pentraxin 3 in SSc-MVECs, thereby confirming the microarray and RT-PCR data at the protein level. Figures 2b,c and 4b,c show that anti-plexin B1 and anti-DSG2 antibodies (each used at 3 μg/ml), respectively, were able to partially inhibit N-MVEC invasion through Matrigel-coated porous filters, and exhibited a strong down-regulation activity in capillary morphogenesis of N-MVECs. Since pent(r)axin 3 was up-regulated in SSc-MVECs, we added SSc-MVEC conditioned medium to N-MVECs for 24 hours, which resulted in a relevant inhibition of both invasion (Figure 3b) and capillary morphogenesis (Figure 3c) of N-MVECs. The effect of SSc-MVEC conditioned medium was much reduced when added to N-MVECs in the presence of 3 μg/ml anti-pent(r)axin antibodies (Figure 3b,c). It is noteworthy that anti-pent(r)axin antibodies exhibited a small inhibiting activity on capillary morphogenesis of N-MVECs independent of the presence of conditioned medium from SSc-MVEC cultures.

## Discussion

To date, this is the first study that compares the differential transcriptosome of MVECs isolated from the skin of normal subjects and from the lesional skin of SSc patients affected by the diffuse form of the disease in the avascular phase.

The number of 6,724 genes that passed the filtering criteria is in agreement with those previously obtained in other human cell lines by microarray analysis [9] and by massively parallel signature sequencing [32]. We have observed that the majority of genes are expressed at similar levels in N-MVECs and SSc-MVECs and that, interestingly, 3.2% of the total filtered transcripts (199 genes) were differentially expressed. Considering the correctness of comparing data limited to transcripts, we show that, in SSc-MVECs, the dysregulation involves only a small number of genes controlling a large number of processes that are critical to the biology of endothelial cells.

Surprisingly, we have found that SSc-MVECs exhibit a pro-angiogenic gene expression pattern (Table 3). In SSc, the angiogenic process is severely impaired in the late phases of the disease [33], independent of the increased levels of circulating Vascular Endothelial Growth Factor (VEGF) and FGF2 [34,35]. Therefore, it is likely that some critical checkpoints in the control of angiogenesis are altered at the MVEC level. Our data indicate down-regulation of PLXNB1, a receptor for semaphorin that tracks the pathway to migrating endothelial cells by activation of the MET oncogene and by stimulating Rho-initiated pathways [13,36,37], of tissue KLK9, KLK11 and KLK12, shown to be required for MVEC migration and proliferation [12], and of DSG2, which is positively involved in homophilic cell-cell interaction [15,38]. At the same time, PTX3, an angiogenesis inhibitor that acts by binding FGF2 [14], is up-regulated (Table 3). We propose to interpret these data as a stabilization of a pro-angiogenic pattern dictated by angiogenesis factors such as VEGF and FGF2 that is blocked or rendered ineffective by the strong down-regulation of the critical adhesion/invasion/proliferation systems and by up-regulation of the angiogenesis inhibitor PTX3 (Table 3). Due to a common technical problem during the spotting procedure, microarray data were not available for matrix metallo-protease-12 (MMP12), which in a previous study on the same SSc-MVECs was found to be up-regulated and responsible for urokinase-type plasminogen activator receptor (uPAR) truncation and subsequent angiogenesis impairment [18]. In this previous work we suggested the possibility that the alterations we observed in SSc-MVECs may initially be stimulated by environment factors and then become the product of the hypoxia-induced selection of an endothelial cell population more suitable to survive in the hypoxic micro-environment typical of the disease. Still, unexpectedly, in SSc-MVECs we have observed an overall up-regulation of many components of several transduction systems. The large majority of such alterations (Table 3, Additional file 5) deal with transduction by small GTPase proteins, which couple signals from ECM molecules to the cell cytoskeleton, inducing alternating states of cell contraction/relaxation [39]. However, up-regulation of Rho GDP dissociation inhibitor beta (ARHGDIB) is likely to provide a critical 'bottle-neck' to GTPase protein transductions by impairing the substitution of GDP with GTP, thus preventing the receptor to enter its activation state [40]. Also, PLXNB1 down-regulation inhibits semaphorin-directed MVEC migration by blunting Rho-initiated pathways [36,37]. Further, an overall impairment to cell proliferation in SSc-MVECs must also be related to down-regulation of MAP4K1, a serine/threonine kinase involved in a variety of cellular signaling cascades [41].

**Figure 5:**
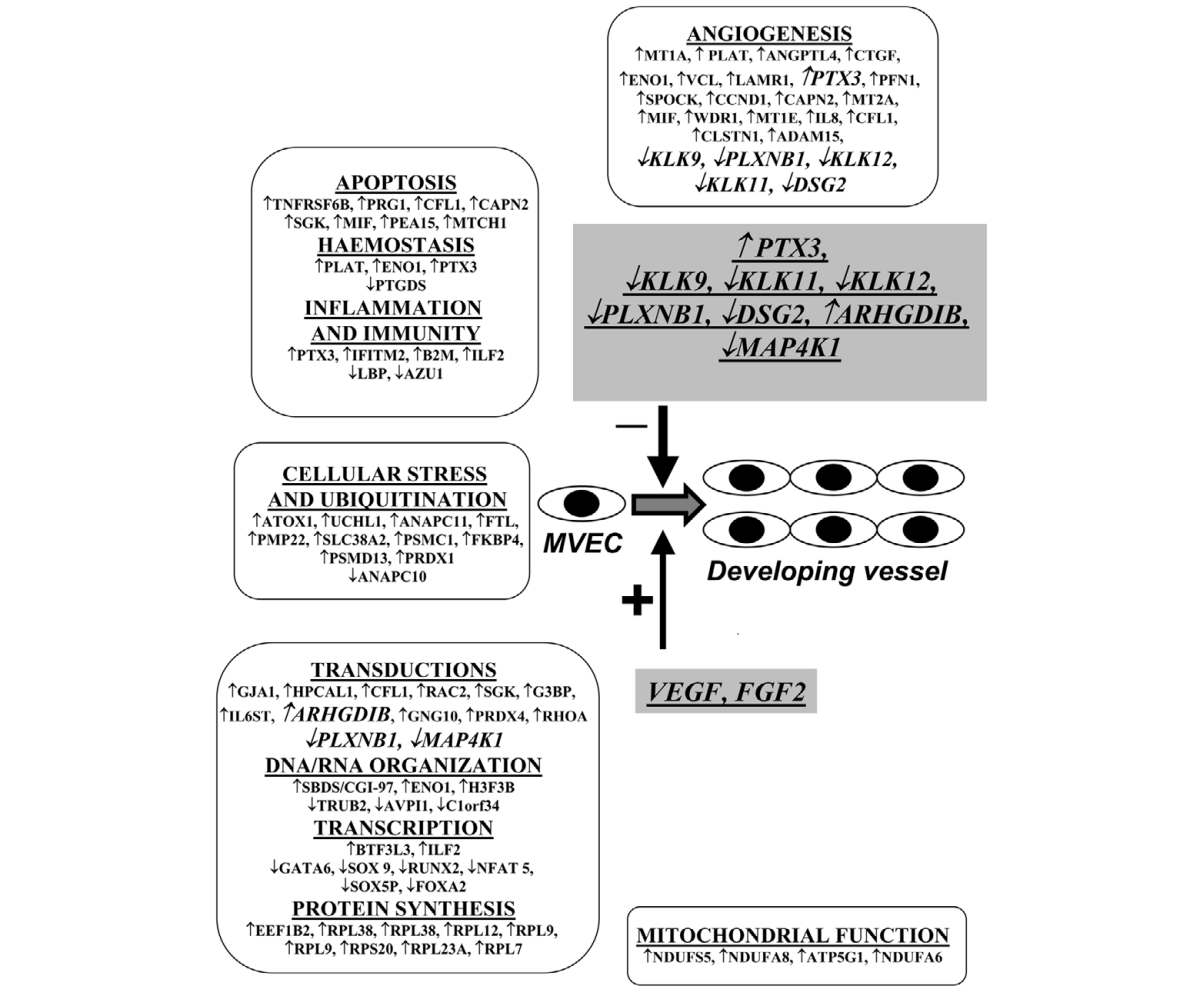
Impairment of angiogenesis in systemic sclerosis. Genes differentially expressed in microvascular endothelial cells (MVECs) from patients with systemic sclerosis (SSc-MVECs) are grouped according to Table 3. Upward and downward arrows indicate up-regulation and down-regulation, respectively, in SSc-MVECs. Differentially expressed genes that we propose to be critical to the altered angiogenic process in SSc are reported in italics in each sub-group. In SSc, the powerful angiogenic instruction of Vascular Endothelial Growth Factor (VEGF) and Fibroblast Growth Factor-2 (FGF2) [34,35] is outstripped by the activity of differentially expressed genes shown as a separate group in the box shaded light grey.

Provided that all the observed alterations in gene expression may be important in the pathogenesis of vascular damage in SSc, our results allow the identification of some genes that may block a correct angiogenesis program in SSc-MVECs. Of particular interest may be genes involved in: MVEC migration, proliferation and adhesion (down-regulated PLXNB1, KLKs, DSG2); inhibition of angiogenesis (up-regulated PTX3); and alteration of signal transduction pathways (up-regulated ARHGDIB, down-regulated PLXNB1, down-regulated MAP4K1) (Figure 5). For six of such genes (PLXNB1, PTX3, KLK9, KLK11, KLK12, DSG2), we have provided evidence of a critical role in the angiogenic process (this work and [12]).

Over-expression of genes of proteasome and ubiquitin pathways suggests the possibility that the observed gene alterations are the effect of a cell adaptation to a particularly hostile environment. It is interesting to underline the induction of 3 metallothionein genes in SSc-MVECs. Metallothioneins belong to a family of stress-induced proteins that regulate Zn and Cu availability and also modulate the amount and activity of NF-_K_B, a transcription factor for genes involved in apoptosis, immune response and inflammation [42].

Since RNA harvested from MVECs was obtained from cells between the fourth and seventh passage, this could raise concerns about MVEC stability and/or possible selection of cells more likely to survive in culture. However, we previously showed that RT-PCR, performed with RNA isolates of cells from single patients at early and late culture passages to validate tissue KLK expression, gave similar results, in agreement with microarray data obtained with pooled RNA [12]. Moreover, the demonstration of down-regulation of KLK9, KLK11, KLK12 and DSG2 in skin biopsies of SSc patients by immunocytochemistry suggests that at least some of the reported alterations pre-exist to the isolation and culture propagation techniques. Nonetheless, a possible selection bias, responsible for some of the reported differences, cannot be ruled out. There is evidence that inference from most genes is not adversely affected by pooling, such that pooling is recommended when fewer than three arrays are used in each condition [43]. Additionally, due to the small number of replicates, we decided to apply the dye swap design to minimize the gene-specific dye bias [44], which is the major source of experimental variability between replicates. In the present study we have applied markedly stringent criteria for feature extraction and data normalization, which could blunt the identification of differentially expressed genes. Nevertheless, we believe that our findings, related to single genes, or classes of genes, may provide hints for future research and are worthy of a more in-depth study to identify possible ways to correct some critical molecular defects and to recover, at least partially, the angiogenic attitude of SSc-MVECs.

## Conclusion

In this study we analyzed gene expression profiles of skin MVECs isolated from healthy subjects and diffuse SSc patients, using a microarray approach and validating data on a series of selected genes by quantitative RT-PCR. Based on gene ontology and other classification criteria, we found that SSc-MVECs, while being unable to perform angiogenesis, over-express a large variety of transcripts positively involved in angiogenesis. However, such a pro-angiogenic pattern of SSc-MVECs is counterbalanced by up-regulation of pent(r)axin 3, a powerful angiogenesis inhibitor, by down-regulation of a few critical pro-angiogenic transcripts (three tissue KLKs, plexin-B1, and DSG), and by alteration of transcripts involved in signal transduction pathways. This different expression profile and identification of a few molecules able to account for impaired angiogenesis in SSc provide the focus for future attempts to correct anti-angiogenesis in SSc.

## Abbreviations

ARHGDIB = Rho GDP dissociation inhibitor beta; CTGF = connective tissue growth factor; DSG = desmoglein; ECM = extracellular matrix; FCS = fetal calf serum; GAPDH = glyceraldehyde-3-phosphate dehydrogenase; GO = gene ontology; IL = interleukin; KLK = kallikrein; LOR = log odds ratio; MVEC = microvascular endothelial cell; PLAU = urokinase type plasminogen activator; RT-PCR = reverse transcription PCR; SD = standard deviation; SSc= systemic sclerosis.

## Competing interests

The authors declare they have no competing interests.

## Authors' contributions

BG and GF carried out critical examinations in this study, coordinated the experiments and drafted the manuscript together with ADR. FM and SS isolated microvascular endothelial cells from skin biopsies, propagated them in culture and carried out RNA extraction for microarray, and RNA amplification for RT-PCR. LR, FP, and IL performed cDNA microarray analyses and RT-PCR studies. AM carried out statistical analysis of microarray and RT-PCR results. BK provided a line of microvascular endothelial cells from a SSc patient and critically revised the manuscript. MC, SG, ADR, and LB carried out the clinical studies of each case, performed targeted biopsies of skin after the informed consent of each patient, and participated in the first phases of microvascular endothelial cells isolation. SB, GFG, MMC, RA, and MDR conceived the study, participated in its design and coordination and wrote the final version of the manuscript. MDR and RA are also the corresponding authors. All authors read and approved the final manuscript. BG, GF authors contributed equally to the results of the present study.

## Supplementary Material

Additional File 1 (**a**) A PDF file showing the 150 most expressed genes in MVECs, independent of their tissue sample origin (normal subjects, SSc patients). Transcripts are listed according to A* = average of log_2 _(RxG) of the two arrays, where R and G represent the fluorescence intensity of Cy5 (red) and Cy3 (green). Grouping of various transcripts allows the identification of the following main categories: genes encoding for cytoskeletal elements; genes encoding for proteins that play a role in regulation of actin polymerization; genes regulating detoxification of heavy metals (such as cadmium, zinc and mercury), essential metal homeostasis, protection against radiation and oxidative damage; genes encoding ribosomal proteins; and miscellanea. (**b**) A PDF file showing the list of all the Gene Ontology (GO) significant terms obtained by the analysis of the 199 differentially expressed genes. This list integrates that shown in Table 1 of the text, where only differentially expressed genes with more than two annotated genes on the array (N > 2) are reported. Click here for file

Additional File 2 A PDF file showing the list of differentially expressed genes involved in angiogenesis. This list integrates that shown in Table 2 of the text, starting from transcripts with LOR >0. Transcripts were sub-divided according to their role in various phases of angiogenesis (migration/invasion, proliferation, adhesion, angiogenesis inhibition). Click here for file

Additional File 3 A PDF file showing the list of differentially expressed genes involved in apoptosis, haemostasis, inflammation and immunity. This list integrates that shown in Table 2 of the text, starting from transcripts with LOR >0. Click here for file

Additional File 4 A PDF file showing the list of differentially expressed genes involved in cellular stress and ubiquitination. This list integrates that shown in Table 2 of the text, starting from transcripts with LOR >0. Click here for file

Additional File 5 A PDF file showing the list of differentially expressed genes involved in stimulus transduction, DNA/RNA organization, and transcription. This list integrates that shown in Table 2 of the text, starting from transcripts with LOR >0. Click here for file

Additional File 6 A PDF file showing the list of differentially expressed genes involved in regulation of protein synthesis and mitochondrial functions. Genes involved in protein synthesis were identified as structural components of the ribosome or as functional regulators of protein synthesis. All of them were up-regulated in SSc-MVECs, as well as transcripts regulating mitochondrial functions. Click here for file

Additional File 7 A PDF file showing the list of differentially expressed genes with unknown function or that cannot be included within a class. Nineteen transcripts were up-regulated and 17 down-regulated in SSc-MVECs. Click here for file

## Figures and Tables

**Table 1:** Time-fold up- or down-expression of genes analyzed by real time PCR in SSc-MVECs versus N-MVECs

Gene name	Time-fold up- or down-expression in SSc-MVECs relative to N-MVECs	
KLK9	10.63 (8.26–13.67)	↓
KLK11	53.07 (41.44–67.98)	↓
KLK12	19.02 (11.61–31.17)	↓
PLXNB1	1.90 (1.41–2.13)	↓
DSG2	35.08 (28.23–43.73)	↓
IL8	2.85 (1.82–4.37)	↑
PLAU	2.44 (1.48–3.65)	↑
PTX3	1.58 (1.29–1.87)	↑
CTGF	1.42 (1.34–1.52)	↑

Upward and downward arrows mean up-regulation and down-regulation in microvascular endothelial cells from patients with systemic sclerosis (SSc-MVECs), respectively. N-MVECs, microvascular endothelial cells from normal subjects.

**Table 2:** List of all the Gene Ontology significant terms with more than two annotated genes on the array (N > 2)

GO category number	GO term	*P *value	n/N	Genes
6412	Protein biosynthesis	<0.001	15/205	EIF4G1(↑), ITGB4BP(↑), LAMR1(↑), RPL7(↑), RPL9(↑), RPL10(↑), RPL12(↑), RPL23A(↑), RPS5(↑), RPS10(↑), RPS20(↑), RPL14(↑), NOLA2(↑), RPL10A(↑), RPL38(↑)
50930	Induction of positive chemotaxis	0.002	2/4	AZU1(↓), IL8(↑)
6878	Copper ion homeostasis	0.002	2/4	ATOX1(↑), MT2A(↑)
6337	Nucleosome disassembly	0.002	2/4	HMGA1(↑), SUPT16H(↑)
6508	Proteolysis and peptidolysis	0.002	13/290	ANPEP(↑), AZU1(↓), CAPN2(↑), NEDD8(↑), PLAT(↑), PLAU, PRSS1(↓), ADAM15(↑), NPEPPS(↑), KLK11(↓), SUPT16H(↑), CASP14(↓), KLK12(↓)
7266	Rho protein signal transduction	0.004	3/18	RHOA(↑), ARHGDIB(↑), CFL1(↑)
1516	Prostaglandin biosynthesis	0.008	2/8	MIF(↑), PTGDS(↓)
30036	Actin cytoskeleton organization and biogenesis	0.010	4/47	RHOA(↑), ARHGDIB(↑), CFL1(↑), PFN1(↑)
42157	Lipoprotein metabolism	0.011	2/9	APOA1(↓), HMGA1(↑)
6928	Cell motility	0.012	6/104	ACTN4(↑), CTGF(↑), IL8(↑), SPOCK(↑), ACTR3(↑), ARPC2(↑)
43065	Positive regulation of apoptosis	0.013	2/10	BCL2L11(↓), MTCH1(↑)
6445	Regulation of translation	0.014	3/28	MKNK2(↑), LAMR1(↑), PPP2CA(↑)
43066	Negative regulation of apoptosis	0.016	2/11	MIF(↑), ANGPTL4(↑)
30155	Regulation of cell adhesion	0.019	2/12	IL8(↑), PPP2CA(↑)
6979	Response to oxidative stress	0.024	3/34	ATOX1(↑), DUSP1(↑), GPX2(↓)
7596	Blood coagulation	0.026	4/61	GP9(↓), PLAT(↑), PLAU(↑), THBD(↑)
6869	Lipid transport	0.026	3/35	APOA1(↓), HMGA1(↑), LBP(↓)
9611	Response to wounding	0.030	2/15	CTGF(↑), MDK(↑)
6355	Regulation of transcription, DNA-dependent	0.037	11/1,042	BTF3L3(↑), RUNX2(↓), ENO1(↓), GATA6(↓), HMGA1(↓), FOXA2(↓), NFKB2(↓), SSRP1(↑), UBE2V1(↑), NFAT5(↓), SIX4(↓)
45941	Positive regulation of transcription	0.047	2/19	GATA6(↓), HMGA1(↑)
7243	Protein kinase cascade	0.047	3/44	MKNK2(↑), STK17A(↓), MAP4K1(↓)
7605	Perception of sound	0.048	4/74	GJA1(↑), PMP22(↑), WDR1(↑), TIMM8B(↑)

*P *values were determined by Fisher's exact test. Genes is a list of differentially expressed genes annotated to the GO terms; upward and downward arrows indicate up- and down-regulation, respectively. GO, gene ontology; n, number of differentially expressed genes annotated to the GO term; N, number of genes represented on the array annotated to the GO term;

**Table 3:** Class distribution of differentially expressed genes with a log odds ratio >1

Gene symbol	Gene title	M
**Angiogenesis**		
MT1A	↑ Metallothionein 1A	3.25
PLAT	↑ Plasminogen activator, tissue	2.23
ANGPTL4	↑ Angiopoietin-like 4	1.97
CTGF	↑ Connective tissue growth factor	1.80
ENO1	↑ Enolase 1	1.67
PTX3	↑ Pentaxin-related gene, rapidly induced by IL-1 beta	1.66
VCL	↑ Vinculin	1.65
LAMR1	↑ Laminin receptor 1	1.63
PFN1	↑ Profilin 1	1.54
MT2A	↑ Metallothionein 2A	1.50
SPOCK	↑ Sparc/osteonectin (testican)	1.46
MT1E	↑ Metallothionein 1E	1.43
CCND1	↑ Cyclin D1	1.40
CFL1	↑ Cofilin 1	1.31
CAPN2	↑ Calpain 2, large subunit	1.28
MIF	↑ Macrophage migration inhibitory factor	1.24
WDR1	↑ WD repeat domain 1	1.21
IL8	↑ IL8	1.18
CLSTN1	↑ Calsyntenin 1	1.07
ADAM15	↑ A disintegrin and metalloproteinase domain 15	1.06
EFEMP1	↑ EGF-containing fibulin-like extracellular matrix protein 1	1.03
KLK9	↓ Kallikrein 9	-1.23
PLXNB1	↓ Plexin B1	-1.19
KLK12	↓ Kallikrein 12	-1.08
KLK11	↓ Kallikrein 11	-1.08
DSG2	↓ Desmoglein 2	-1.04
		
**Apoptosis**		
TNFRSF6B	↑ Tumor necrosis factor receptor superfamily, member 6b, decoy	2.20
PRG1	↑ Proteoglycan 1, secretory granule	1.32
CFL1	↑ Cofilin 1	1.31
CAPN2	↑ Calpain 2, (m/II) large subunit	1.28
SGK	↑ Serum/glucocorticoid regulated kinase	1.27
MIF	↑ Macrophage migration inhibitory factor	1.24
PEA15	↑ Phosphoprotein enriched in astrocytes 15	1.23
MTCH1	↑ Mitochondrial carrier homolog 1	1.08
		
**Haemostasis, inflammation and immunity**		
PLAT	↑ Plasminogen activator, tissue	2.23
ENO1	↑ Enolase 1	1.67
PTX3	↑ Pentaxin-related gene	1.66
IFITM2	↑ Interferon induced transmembrane protein 2	1.27
B2M	↑ Beta-2-microglobulin	1.26
ILF2	↑ Interleukin enhancer binding factor 2	1.15
LBP	↓ Lipopolysaccharide binding protein	-1.77
PTGDS	↓ Prostaglandin D2 synthase 21 kDa	-1.14
AZU1	↓ Azurocidin 1	-1.02
		
**Stress/ubiquitination**		
ATOX1	↑ ATX1 antioxidant protein 1 homolog	1.51
UCHL1	↑ Ubiquitin carboxy-terminal esterase L1	1.33
ANAPC11	↑ Anaphase promoting complex subunit 11 homolog	1.28
FTL	↑ Ferritin, light polypeptide	1.25
PMP22	↑ Peripheral myelin protein 22	1.19
SLC38A2	↑ Solute carrier family 38, member 2	1.14
PSMC1	↑ Proteasome (prosome, macropain) 26S subunit, ATPase, 1	1.12
FKBP4	↑ FK506 binding protein 4	1.12
PSMD13	↑ Proteasome (prosome, macropain) 26S subunit, non-ATPase, 13	1.03
PRDX1	↑ Peroxiredoxin 1.	1.02
ANAPC10	↓ Anaphase promoting complex subunit 10	-1.22
		
**Transduction**		
GJA1	↑ Gap junction protein, alpha 1, 43 kDa (connexin 43)	1.39
HPCAL1	↑ Hippocalcin-like 1	1.36
CFL1	↑ Cofilin 1	1.31
RAC2	↑ Ras-related C3 botulinum toxin substrate 2	1.30
SGK	↑ Serum/glucocorticoid regulated kinase	1.27
IL6ST	↑ Interleukin 6 signal transducer (gp130, oncostatin M receptor)	1.15
ARHGDIB	↑ Rho GDP dissociation inhibitor beta	1.10
GNG10	↑ Guanine nucleotide binding protein, gamma 10	1.05
PRDX4	↑ Peroxiredoxin 4	1.05
RHOA	↑ Ras homolog gene family, member A	1.02
PLXNB1	↓ Plexin B1	-1.19
MAP4K1	↓ Mitogen-activated protein kinase/kinase/kinase/kinase 1	-1.05
		
**DNA/RNA organization**		
SBDS/CGI97	↑ Shwachman-Bodian-Diamond syndrome	2.14
ENO1	↑ Enolase 1	1.67
H3F3B	↑ H3 histone, family 3B	1.37
TRUB2	↓ TruB pseudouridine synthase homolog 2	-1.23
AVPI1	↓ Arginine vasopressin-induced 1	-1.12
C1orf34	↓ Chromosome 1 open reading frame 34	-1.05
		
**Transcription**		
BTF3L3	↑ Basic transcription factor 3, like 3	1.21
ILF2	↑ Interleukin enhancer binding factor 2	1.15
GATA6	↓ GATA binding protein 6	-2.19
SOX9	↓ SRY (sex determining region Y)-box 9	-1.99
RUNX2	↓ Runt-related transcription factor 2	-1.16
NFAT5	↓ Nuclear factor of activated T-cells 5	-1.14
SOX5P	↓ SRY (sex determining region Y)-box 5	-1.12
FOXA2	↓ Forkhead box A2	-1.10

All genes with a log odds ratio >0 were considered significantly down-regulated (M < 0) or up-regulated (M > 0). The table reports only genes with M > 1 and M < 1. Upward and downward arrows indicate up- and down-regulation, respectively.
