# Supplementary material for: A model of anti-angiogenesis: differential transcriptosome profiling of microvascular endothelial cells from diffuse systemic sclerosis patients
Source: Arthritis Res Ther. 2006 Jul 19;8(4):R115. doi: 10.1186/ar2002 (PMC1779372; doi:10.1186/ar2002)
Supplement: Additional File 1 — (a) A PDF file showing the 150 most expressed genes in MVECs, independent of their tissue sample origin (normal subjects, SSc patients). Transcripts are listed according to A* = average of log2 (RxG) of the two arrays, where R and G represent the fluorescence intensity of Cy5 (red) and Cy3 (green). Grouping of various transcripts allows the identification of the following main categories: genes encoding for cytoskeletal elements; genes encoding for proteins that play a role in regulation of actin polymerization; genes regulating detoxification of heavy metals (such as cadmium, zinc and mercury), essential metal homeostasis, protection against radiation and oxidative damage; genes encoding ribosomal proteins; and miscellanea. (b) A PDF file showing the list of all the Gene Ontology (GO) significant terms obtained by the analysis of the 199 differentially expressed genes. This list integrates that shown in Table 1 of the text, where only differentially expressed genes with more than two annotated genes on the array (N > 2) are reported. [file ar2002-S1.pdf]

Additional file 1A. The 150 most expressed genes in MVEC

Symbol	GenBank	M	LOR	A*	Gene Name
**VIM**	AL133415	1.29E-01	-4.43378	14.31794	vimentin
**ACTB**	NM_001101	1.18E+00	-0.22203	14.10054	actin, beta
**TMSB4X**	M17733	-1.81E-01	-4.22147	14.07129	thymosin, beta 4, X chromosome
**MT1B**	M13485	1.21E+00	0.16528	13.80912	metallothionein 1B (functional)
**MT2A**	V00594	1.50E+00	2.439238	13.72704	metallothionein 2A
**LOC390363**	U85977	1.03E-01	-3.95315	13.55643	similar to Chloride intracellular channel protein 1 (Nuclear chloride ion channel 27)
**RPL37A**	L06499	8.28E-01	-1.83686	13.42279	ribosomal protein L37a
**RPL19**	X63527	5.63E-01	-2.92867	13.40577	ribosomal protein L19
**GAPD**	U34995	7.08E-01	-2.26319	13.23161	glyceraldehyde-3-phosphate dehydrogenase
**MT1E**	M10942	1.43E+00	2.208412	13.22502	metallothionein 1E (functional)
**DIO1**		-1.75E-01	-3.62832	13.14651	deiodinase, iodothyronine, type I
**TUBA3**	X01703	4.82E-01	-3.01534	13.14277	Tubulin, alpha, brain-specific
**RPLP1**	M17886	5.85E-01	-2.66302	13.10831	ribosomal protein, large, P1
**FLJ20464**	AK000471	9.52E-02	-3.64829	13.07524	hypothetical protein FLJ20464
**RPL15**	L25899	4.45E-01	-3.06654	13.06266	ribosomal protein L15
**NPHP1**	NM_000272	-7.84E-02	-3.63871	13.04624	nephronophthisis 1 (juvenile)
**NDUFB7**	M33374	-3.71E-01	-3.21526	13.00654	NADH dehydrogenase (ubiquinone) 1 beta subcomplex, 7 (18kD, B18)
**UBBP1**	X04801	4.13E-01	-2.9906	12.81011	Homo sapiens UBBP1 pseudogene for ubiquitin UBB
**RNU35**	X56932	6.09E-01	-2.37615	12.78982	RNA, U35 small nucleolar
**RPS18**	X69150	6.84E-01	-2.0199	12.68465	ribosomal protein S18
**RPL10**	M64241	1.20E+00	0.819548	12.64658	ribosomal protein L10
**GNAI2**	X04828	3.38E-01	-3.0255	12.59341	guanine nucleotide binding protein (G protein), alpha inhibiting activity polypeptide 2
**FLVCR**	AK001419	1.93E-01	-3.2548	12.58521	FLVCR protein
**LOC440551**	AK000309	6.17E-01	-2.21199	12.5761	similar to large low complexity protein with proline/alanine-rich repeat
**DMC1**	D64108	1.09E-01	-3.3138	12.55695	DMC1 (dosage suppressor of mck1, yeast homolog) meiosis-specific homologous recombination
**TSRC1**	AL122085	-4.33E-02	-3.33746	12.54594	thrombospondin repeat containing 0
**PTPRO**	NM_002848	-1.01E+00	-0.26014	12.47969	protein tyrosine phosphatase, receptor type, O
**RPS24**	M31520	7.40E-01	-1.65657	12.47957	ribosomal protein S24
**RPLP2**	M17887	5.05E-01	-2.53073	12.47915	ribosomal protein, large P2
**GP2**	U36221	-9.31E-02	-3.21777	12.38501	glycoprotein 2 (zymogen granule membrane)

**C6orf106**	AL031577	4.84E-01	-2.53238	12.38065	chromosome 6 open reading frame 105
**LGALS1**	Z83844	4.78E-01	-2.52669	12.34071	lectin, galactoside-binding, soluble, 1 (galectin 1)
**RPLP2**	AB002533	4.81E-01	-2.51417	12.33565	ribosomal protein, large P1
**PPEF2**	AF023456	8.09E-02	-3.18095	12.31411	protein phosphatase, EF hand calcium-binding domain 2
**UBA52**	X56997	6.64E-01	-1.86047	12.29311	ubiquitin A-52 residue ribosomal protein fusion product 1
**GNAS**	AF105253	2.74E-01	-2.91532	12.21806	GNAS complex locus
**PKHD1L1**	AL133640	2.60E-01	-2.93726	12.21609	polycystic kidney and hepatic disease 1 (autosomal recessive)-like 0
**TMSB10**	S54005	1.62E-01	-3.0567	12.20828	thymosin, beta 10
**ITGA5**	X06256	2.61E-01	-2.91745	12.18652	integrin, alpha 5 (fibronectin receptor, alpha polypeptide)
**RPL4**	D23660	3.05E-01	-2.81461	12.14268	ribosomal protein L4
**EEF2**	Z11692	4.23E-01	-2.52486	12.09156	eukaryotic translation elongation factor 2
**GNB2L1**	M24194	6.99E-01	-1.59973	12.09083	guanine nucleotide binding protein (G protein), beta polypeptide 2-like 1
**GRIPAP1**	AB032993	3.57E-01	-2.67838	12.08822	GRIP1 associated protein 0
**LMO6**	AJ011654	-1.05E-01	-3.01902	12.06748	LIM domain only 6
**PLP2**	U93305	5.12E-02	-3.04421	12.06745	proteolipid protein 2 (colonic epithelium-enriched)
**MT1A**	K01383	3.25E+00	23.81327	12.05785	metallothionein 1A (functional)
**CDC14B**	NM_003671	-1.73E-01	-2.95117	12.04965	CDC14 (cell division cycle 14, S. cerevisiae) homolog B
**CFL1**	X95404	1.31E+00	1.992094	12.04704	cofilin 1 (non-muscle)
**ATF5**	AB021663	2.50E-01	-2.83982	12.02971	activating transcription factor 5
**RPL17**	X53777	5.87E-01	-1.9919	12.02814	ribosomal protein L17
**IGFBP7**	S75725	8.04E-01	-1.09213	12.02718	insulin-like growth factor binding protein 7
**CLNS1A**	X91788	-1.06E-01	-2.9942	12.02712	chloride channel, nucleotide-sensitive, 1A
**RPL9**	U09953	1.41E+00	2.758467	12.023	ribosomal protein L9
**APLP2**	S60099	3.38E-01	-2.67639	12.01801	amyloid beta (A4) precursor-like protein 2
**RPL38**	Z26876	1.69E+00	5.196313	11.98611	ribosomal protein L38
**CSTB**	AF208234	2.37E-01	-2.8227	11.96715	cystatin B (stefin B)
**RPL30**	L05095	8.62E-01	-0.77291	11.96244	ribosomal protein L30
**ZBTB16**	Z19002	1.92E-02	-2.98751	11.96028	zinc finger and BTB domain containing 15
**LOC150400**	Z82202	3.85E-01	-2.52463	11.93325	similar to 60S ribosomal protein L34
**RPS20**	L06498	1.34E+00	2.334977	11.90106	ribosomal protein S20
**FTHP1**	J04755	-4.32E-02	-2.94678	11.89869	ferritin, heavy polypeptide pseudogene 1
**PRSS23**	AF015287	3.24E-01	-2.61988	11.87303	~~protease, serine, 22~~

**SERPINC1**	L00190	6.54E-02	-2.91654	11.8595	serine (or cysteine) proteinase inhibitor, clade C (antithrombin), member
**CCND1**	X59798	1.41E+00	2.836313	11.84522	cyclin D1 (PRAD1: parathyroid adenomatosis 1)
**EEF1G**	X63526	3.56E-01	-2.53091	11.83317	eukaryotic translation elongation factor 1 gamma
**RPS5**	U14970	1.07E+00	0.488473	11.81729	ribosomal protein S5
**LOC442257**	AL035603	-7.34E-02	-2.88309	11.80772	similar to ribosomal protein S4, X-linked
**ARFGAP1**	AK001629	1.69E-01	-2.79351	11.77355	ADP-ribosylation factor GTPase activating protein 0
**DSCR4**	AB000099	6.40E-02	-2.86389	11.76779	Down syndrome critical region gene 4
**FDPS**	D14697	2.38E-01	-2.70054	11.76038	farnesyl diphosphate synthase (farnesyl pyrophosphate synthetase, dimethylallyltranstransferase, ger
**TAF2H**	U13991	-8.73E-02	-2.8484	11.75941	TATA box binding protein (TBP)-associated factor, RNA polymerase II, H, 30kD
**RPL12**	AF037643	1.57E+00	4.26808	11.75797	ribosomal protein L12
**HSPB1**	Z23090	1.11E-01	-2.83017	11.75292	heat shock 27kD protein 1
**DES**	M63391	1.59E-01	-2.79083	11.75214	desmin
**TIMELESS**	AF098162	-1.16E-01	-2.82395	11.74762	timeless (Drosophila) homolog
**DNAJC8**	AF083190	-5.95E-01	-1.79847	11.74489	DnaJ (Hsp40) homolog, subfamily C, member 7
**ARHGAP1**	U02570	1.85E-01	-2.75969	11.74482	Rho GTPase activating protein 1
**NFE2L1**	L24123	2.16E-02	-2.8613	11.74424	nuclear factor (erythroid-derived 2)-like 1
**ATP5D**	X63422	3.00E-02	-2.8574	11.73974	ATP synthase, H+ transporting, mitochondrial F1 complex, delta subunit
**BIN2**	NM_016187	1.37E-01	-2.79762	11.7301	bridging integrator 2
**VDR**	J03258	9.97E-02	-2.82114	11.72421	vitamin D (1,25- dihydroxyvitamin D3) receptor
**WNT8B**	Y11094	-2.62E-03	-2.84104	11.70652	wingless-type MMTV integration site family, member 8B
**ZNF536**	AB002388	-4.53E-02	-2.83414	11.70528	zinc finger protein 535
**RPL31**	X15940	4.02E-01	-2.34956	11.70076	ribosomal protein L31
**ZNF354A**	AF116030	-2.43E-02	-2.82864	11.68795	zinc finger protein 354A
**HOXD8**	X15507	9.07E-02	-2.80423	11.68565	Human Hox5.4 gene fragment
**FHR-4**	X98337	4.06E-01	-2.32182	11.66667	complement factor H-related 4
**SQSTM1**	U46751	9.39E-01	-0.19429	11.66064	sequestosome 1
**MPHOSPH9**	X98258	2.99E-01	-2.54434	11.65995	M-phase phosphoprotein 9
**APOBEC1**	AB009426	3.93E-02	-2.80947	11.65938	apolipoprotein B mRNA editing enzyme, catalytic polypeptide 1
**NOTCH2**	AL133036	9.41E-02	-2.7801	11.64665	hypothetical protein DKFZp434N181
**GCK**	M90299	-2.71E-01	-2.58129	11.6394	glucokinase (hexokinase 4,

					maturity onset diabetes of the young 2)
**LYZ**	AL157462	9.26E-02	-2.77269	11.63214	lysozyme (renal amyloidosis)
**COCH**	AF006740	-3.70E-02	-2.79293	11.62937	coagulation factor C (Limulus polyphemus) homology (cochlin)
**AES**	NM_001130	1.05E-01	-2.75733	11.61848	amino-terminal enhancer of split
**ZW10**	U54996	2.50E-01	-2.5978	11.60961	ZW10 (Drosophila) homolog, centromere/kinetochore protein
**G1P3**	AB019565	2.48E-01	-2.59679	11.6031	interferon, alpha-inducible protein (clone IFI-6-16)
**RPS27A**	S79522	7.23E-01	-1.21434	11.5986	ribosomal protein S27a
**B3GAT3**	AB009598	-1.55E-01	-2.69265	11.57267	beta-1,3-glucuronyltransferase 3 (glucuronosyltransferase I)
**SEPW1**	U67171	-4.29E-02	-2.75752	11.56919	selenoprotein W, 1
**F2R**	NM_001992	-5.33E-01	-1.90732	11.56894	coagulation factor II (thrombin) receptor
**RNF19**	AL122096	2.30E-04	-2.75786	11.5599	ring finger protein 18
**PTX3**	M31166	1.66E+00	5.192572	11.54302	pentaxin-related gene, rapidly induced by IL-1 beta
**RORA**	U04898	1.38E-01	-2.68997	11.54088	RAR-related orphan receptor A
**CDK5RAP1**	NM_016082	-1.83E-01	-2.64588	11.53977	CDK5 regulatory subunit associated protein 0
**ZC3HAV1**	AF138863	-9.72E-02	-2.71719	11.5383	Homo sapiens clone FLB6421 mRNA sequence
**GJB1**	X04325	-1.31E-01	-2.69372	11.53761	gap junction protein, beta 1, 32kD (connexin 32, Charcot-Marie-Tooth neuropathy, X-linked)
**ZNF32**	U69645	3.01E-02	-2.73274	11.51994	zinc finger protein 32 (KOX 30)
**MAPKBP1**	AB011168	4.67E-01	-2.07773	11.51681	mouse mitogen-activated protein kinase binding protein 1-like
**PSG9**	M94890	4.75E-02	-2.72164	11.50735	pregnancy specific beta-1-glycoprotein 9
**CRAT**	NM_004003	-6.76E-02	-2.71446	11.50705	carnitine acetyltransferase
**SRGAP2**	AB007925	-6.60E-02	-2.7109	11.49949	KIAA0456 protein
**UBE2C**	U73379	-1.77E-01	-2.62813	11.49741	ubiquitin-conjugating enzyme E2C
**ZNF157**	U28687	3.62E-01	-2.32466	11.49357	zinc finger protein 157 (HZF22)
**CD63**	X62654	4.93E-01	-1.98734	11.48708	CD63 antigen (melanoma 1 antigen)
**PSMD4**	U51007	2.85E-01	-2.47074	11.48349	proteasome (prosome, macropain) 26S subunit, non-ATPase, 4
**RPS9P2**	AL080243	5.84E-01	-1.68769	11.47503	ribosomal protein S9 pseudogene 1
**GNRH2**	AF036329	-6.55E-02	-2.69639	11.47298	gonadotropin-releasing hormone 2
**RPL8**	Z28407	1.08E-01	-2.67412	11.47294	ribosomal protein L8
**KOC1**	AF117108	-1.65E-01	-2.62629	11.47082	IGF-II mRNA-binding protein 3
**ATP5E**	NM_006886	1.57E-01	-2.63352	11.46999	ATP synthase, H+ transporting, mitochondrial F1 complex, epsilon
**IL1RL1**	AB012701	5.53E-02	-2.69781	11.46886	interleukin 1 receptor-like 1
**F9**	K02402	-7.02E-02	-2.68701	11.45954	coagulation factor IX (plasma thromboplastic component,

					Christmas disease, hemophilia B)
**GRN**	AF055008	5.19E-01	-1.89106	11.45651	granulin
**OPTC**	AJ133790	-7.17E-02	-2.68359	11.45457	opticin
**PIB5PA**	U45975	-2.38E-01	-2.52767	11.45321	phosphatidylinositol (4,5) bisphosphate 5-phosphatase, A
**ITGA6**	X53586	3.07E-01	-2.40244	11.43262	integrin, alpha 6
**ITGA3**	M59911	4.79E-01	-1.99617	11.43078	integrin, alpha 3 (antigen CD49C, alpha 3 subunit of VLA-3 receptor)
**POLG**	X98093	-3.32E-02	-2.68179	11.42915	polymerase (DNA directed), gamma
**C21orf33**	U53003	2.63E-02	-2.68201	11.42731	chromosome 21 open reading frame 32
**NUCB2**	X76732	1.79E-01	-2.58613	11.42601	nucleobindin 2
**RARSL**		-5.40E-02	-2.66954	11.41683	arginyl-tRNA synthetase-like
**VBP1**	U96759	8.64E-02	-2.65338	11.41246	von Hippel-Lindau binding protein 1
**TXNRD1**	X91247	2.63E-01	-2.46689	11.41195	thioredoxin reductase 1
**LILRA4**	AF041261	-1.27E-01	-2.62569	11.40914	leukocyte immunoglobulin-like receptor, subfamily A (with TM domain), member 3
**OAS1**	NM_016816	-2.35E-01	-2.503	11.40116	2',5'-oligoadenylate synthetase 1
**FCGR3A**	J04162	-2.26E-02	-2.66714	11.39924	Fc fragment of IgG, low affinity IIIa, receptor for (CD16)
**IGFBP4**	U20982	4.69E-01	-2.00602	11.39688	insulin-like growth factor-binding protein 4
**GPD1L**	D42047	2.17E-01	-2.52475	11.39572	glycerol-3-phosphate dehydrogenase 1-like
**SLC12A1**	U58130	-1.10E-01	-2.62765	11.39142	solute carrier family 12 (sodium/potassium/chloride transporters), member 1
**PMS2L6**	D38503	2.75E-02	-2.66104	11.38947	postmeiotic segregation increased 2-like 6
**IL11**	X58377	1.45E-01	-2.59795	11.38598	interleukin 11
**GJA4**	M96789	3.95E-02	-2.65495	11.38279	gap junction protein, alpha 4, 37kD (connexin 37)
**ZP2**	M90366	-1.93E-01	-2.54657	11.38215	zona pellucida glycoprotein 2 (sperm receptor)
**MASA**	AL110167	-4.37E-02	-2.65312	11.38136	E-1 enzyme
**RNF31**	AK000973	-9.53E-02	-2.63052	11.37966	ring finger protein 30
**SSX3**	U90840	-2.02E-01	-2.5346	11.37918	synovial sarcoma, X breakpoint 3
**MMS19L**	AF007151	2.74E-01	-2.43115	11.37858	MMS19-like (MET18 homolog, S. cerevisiae)
**MYBPC3**	Y10129	7.23E-02	-2.64083	11.37721	myosin-binding protein C, cardiac
**T54**	U66359	1.13E-01	-2.61667	11.37439	T54 protein

A*= average of $\log_2\sqrt{(RxG)}$ of the two arrays; M = differential expression ratio after dye-swap normalization; LOR=log odds ratio: all genes with LOR $\geq$ 0 were considered significantly down-regulated (M<0) or up-regulated (M>0)

Among the first 150 most strongly expressed genes in MVEC obtained from both SSc and control subjects were: a) genes encoding for cytoskeletal elements such as vimentin (VIM), beta actin (ACTB), tubulin alpha 3 (TUBA3), desmin (DES); b) genes encoding proteins which play a role in regulation of actin polymerization: thymosin beta 4, X-linked (TMSB4X), nephronophthisis 1 (NPHP1), thymosin beta 10 (TMSB10), cofilin 1 (CFL1), heat shock 27kDa protein 1 (HSPB1); c) genes regulating detoxification of heavy metals (such as cadmium, zinc and mercury), essential metal homeostasis, protection against radiation and oxidative damage, thereby contributing to cellular proliferation and apoptosis: metallothionein 1B (MT1B), 2A (MT2A), 1E (MT2E) and 1A (MT1A); d) genes encoding ribosomal proteins (RPL37A, RPL19, RPLP1, RPL15, RPL13A, RPS18, RPL10, RPS24, RPLP2, RPL4, RPL17, RPL9, RPL38, RPL30, RPS20, RPS5, RPL12, RPL31, RPS27A, RPS9P2, RPL8); e) glyceraldehyde-3-phosphate dehydrogenase gene (GAPDH); f) ubiquitin B pseudogene 1 (UBBP1), ubiquitin A-52 residue ribosomal protein fusion product 1 (UBA52); g) genes involved in signal transduction such as guanine nucleotide binding protein (G protein), alpha inhibiting activity polypeptide 2 (GNAI2), galectin 1 (LGALS1), GNAS complex locus (GNAS), guanine nucleotide binding protein (G protein) beta polypeptide 2-like 1 (GNB2L1), GRIP1 associated protein 1 (GRIPAP1), amyloid beta (A4) precursor-like protein 2 (APLP2), ADP-ribosylation factor GTPase activating protein 1 (ARFGAP1), Rho GTPase activating protein 1 (ARHGAP1), sequestosome 1 (SQSTM1); h) genes encoding blood coagulation proteins such as serine (or cysteine) proteinase inhibitor clade C (antithrombin) member 1 (ATIII or SERPINC1), coagulation factor II (thrombin) receptor (F2R), coagulation factor IX (F9) ; and i) glycoprotein 2 (GP2), integrin alpha 5 (fibronectin receptor alpha polypeptide) (ITGA5), insulin-like growth factor binding protein 7 (MAC25 or IGFBP7), serine protease 23 (PRSS23), cyclin D1 (CCND1), vitamin D receptor (VDR).

Additional file 1B. List of all the Gene Ontology (GO) significant terms obtained by the analysis of the 199 differentially expressed genes

GO category number	GO TERM	P	n/N	Genes
**6412**	protein biosynthesis	<0.001	15/205	EIF4G1(↑), ITGB4BP(↑), LAMR1(↑), RPL7(↑), RPL9(↑), RPL10(↑), RPL12(↑), RPL23A(↑), RPS5(↑), RPS10(↑), RPS20(↑), RPL14(↑), NOLA2(↑), RPL10A(↑), RPL38(↑)
**50930**	induction of positive chemotaxis	0.002	2/4	AZU1(↓), IL8(↑)
**6878**	copper ion homeostasis	0.002	2/4	ATOX1(↑), MT2A(↑)
**6337**	nucleosome disassembly	0.002	2/4	HMGA1(↑), SUPT16H(↑)
**6508**	proteolysis and peptidolysis	0.002	13/290	ANPEP(↑), AZU1(↓), CAPN2(↑), NEDD8(↑), PLAT(↑), PLAU(↑), PRSS1(↓), ADAM15(↑), NPEPPS(↑), KLK11(↓), SUPT16H(↑), CASP14(↓), KLK12(↓)
**7266**	Rho protein signal transduction	0.004	3/18	RHOA(↑), ARHGDIB(↑), CFL1(↑)
**1516**	prostaglandin biosynthesis	0.008	2/8	MIF(↑), PTGDS(↓)
**30036**	actin cytoskeleton organization and biogenesis	0.010	4/47	RHOA(↑), ARHGDIB(↑), CFL1(↑), PFN1(↑)
**42157**	lipoprotein metabolism	0.011	2/9	APOA1(↓), HMGA1(↑)
**6928**	cell motility	0.012	6/104	ACTN4(↑), CTGF(↑), IL8(↑), SPOCK(↑),ACTR3(↑),ARPC2(↑)
**43065**	positive regulation of apoptosis	0.013	2/10	BCL2L11(↓), MTCH1(↑)
**6445**	regulation of translation	0.014	3/28	MKNK2(↑), LAMR1(↑), PPP2CA(↑)
**43066**	negative regulation of apoptosis	0.016	2/11	MIF(↑), ANGPTL4(↑)
**45869**	negative regulation of retroviral genome replication	0.018	1/1	APOBEC3B(↓)
**45187**	regulation of circadian sleep/wake cycle, sleep	0.018	1/1	PTGDS(↓)
**45610**	regulation of hemocyte differentiation	0.018	1/1	MAP4K1(↓)
**1774**	microglial cell activation	0.018	1/1	AZU1(↓)
**8347**	glial cell migration	0.018	1/1	AZU1(↓)
**42117**	monocyte activation	0.018	1/1	AZU1(↓)
**45123**	cellular extravasation	0.018	1/1	AZU1(↓)
**45348**	positive regulation of MHC class II biosynthesis	0.018	1/1	AZU1(↓)

**50725**	positive regulation of interleukin-1 beta biosynthesis	0.018	1/1	AZU1(↓)
**50754**	positive regulation of fractalkine biosynthesis	0.018	1/1	AZU1(↓)
**50829**	defense response to Gram-negative bacteria	0.018	1/1	AZU1(↓)
**51005**	negative regulation of lipoprotein lipase activity	0.018	1/1	ANGPTL4(↑)
**45091**	regulation of retroviral genome replication	0.018	1/1	IL8(↑)
**48268**	clathrin cage assembly	0.018	1/1	AP2S1(↑)
**7252**	I-kappaB phosphorylation	0.018	1/1	PRDX4(↑)
**6200**	ATP catabolism	0.018	1/1	ACLY(↑)
**15936**	coenzyme A metabolism	0.018	1/1	ACLY(↑)
**42346**	positive regulation of NF-kappaB-nucleus import	0.018	1/1	RHOA(↑)
**16558**	peroxisome matrix protein import	0.018	1/1	PEX10(↑)
**30968**	unfolded protein response	0.018	1/1	VCP(↑)
**30970**	retrograde protein transport, ER to cytosol	0.018	1/1	VCP(↑)
**45184**	establishment of protein localization	0.018	1/1	VCP(↑)
**30325**	adrenal gland development	0.018	1/1	MDK(↑)
**30155**	regulation of cell adhesion	0.019	2/12	IL8(↑), PPP2CA(↑)
**6979**	response to oxidative stress	0.024	3/34	ATOX1(↑),DUSP1(↑),GPX2(↓)
**7596**	blood coagulation	0.026	4/61	GP9(↓), PLAT(↑), PLAU(↑), THBD(↑)
**6869**	lipid transport	0.026	3/35	APOA1(↓), HMGA1(↑), LBP(↓)
**9611**	response to wounding	0.030	2/15	CTGF(↑), MDK(↑)
**30071**	regulation of mitotic metaphase/anaphase transition	0.036	1/2	ANAPC10(↓)
**42535**	positive regulation of tumor necrosis factor-alpha biosynthesis	0.036	1/2	AZU1(↓)
**9267**	cellular response to starvation	0.036	1/2	ANGPTL4(↓)
**45834**	positive regulation of lipid metabolism	0.036	1/2	ANGPTL4(↓)
**43030**	regulation of macrophage activation	0.036	1/2	MIF(↑)
**15988**	energy coupled proton transport, against electrochemical gradient	0.036	1/2	ATP6V1B2(↑)
**6101**	citrate metabolism	0.036	1/2	ACLY(↑)
**6345**	loss of chromatin silencing	0.036	1/2	HMGA1(↑)
**6122**	mitochondrial electron	0.036	1/2	UQCRH(↑)

	transport, ubiquinol to cytochrome c			
**45839**	negative regulation of mitosis	0.036	1/2	AKIP(↑)
**6355**	regulation of transcription, DNA-dependent	0.037	11/1042	BTF3L3(↑), RUNX2(↓), ENO1(↓), GATA6(↓), HMGA1(↓), FOXA2(↓), NFKB2(↓), SSRP1(↑), UBE2V1(↑), NFAT5(↓) ,SIX4(↓)
**45941**	positive regulation of transcription	0.047	2/19	GATA6(↓), HMGA1(↑)
**7243**	protein kinase cascade	0.047	3/44	MKNK2(↑), STK17A(↓), MAP4K1(↓)
**7605**	perception of sound	0.048	4/74	GJA1(↑), PMP22(↑), WDR1(↑), TIMM8B(↑)

P = P value by Fisher's exact test; n = Number of differentially expressed genes annotated to the GO term; N = Number of genes represented on the array annotated to the GO term; Genes = list of differentially expressed genes annotated to the GO term. Upward and downward arrows indicate up-regulated and down-regulated genes in SSc-MVEC, respectively.
