# Supplementary material for: A model of anti-angiogenesis: differential transcriptosome profiling of microvascular endothelial cells from diffuse systemic sclerosis patients
Source: Arthritis Res Ther. 2006 Jul 19;8(4):R115. doi: 10.1186/ar2002 (PMC1779372; doi:10.1186/ar2002)
Supplement: Additional File 2 — A PDF file showing the list of differentially expressed genes involved in angiogenesis. This list integrates that shown in Table 2 of the text, starting from transcripts with LOR >0. Transcripts were sub-divided according to their role in various phases of angiogenesis (migration/invasion, proliferation, adhesion, angiogenesis inhibition). [file ar2002-S2.pdf]

Additional file 2. Differentially expressed genes involved in angiogenesis.

Gene and biological function	Symbol	GenBank	Unigene	M	LOR
**CELL MIGRATION/INVASION**					
↑**(1) Metallothionein 1A (functional)**<br>*Regulation of Zn and Cu availability*	**MT1A**	K01383	440939	3.25	23.81
↑**(2) Plasminogen activator, tissue**<br>*Degradation of provisional fibrin matrix*	**PLAT**	M15518	491582	2.23	11.50
↑**(6) Angiopoietin-like 4**<br>*Positive regulation of angiogenesis*	**ANGPTL4**	NM_016109	9613	1.97	8.75
↑**(8) Connective tissue growth factor**<br>*Positive regulation of angiogenesis, ECM synthesis*	**CTGF**	X78947	410037	1.80	6.71
↑**(10) Enolase 1, (alpha)**<br>*Binding of plasminogen, ECM degradation*	**ENO1**	M14328	517145	1.67	5.79
↑**(19) Metallothionein 2A**<br>*Regulation of Zn and Cu availability*	**MT2A**	V00594	418241	1.50	2.44
↑**(20) Sparc/osteonectin, cwcv and kazal-like domains proteoglycan (testican)**<br>*Protease inhibition, ECM stabilization*	**SPOCK**	AF231124	124611	1.46	3.15
↑**(21) Metallothionein 1E (functional)**<br>*Regulation of Zn and Cu availability*	**MT1E**	M10942	534330	1.43	2.20
↑**(23) Cyclin D1 (PRAD1: parathyroid adenomatosis 1)**<br>*Cytokinesis, invasion*	**CCND1**	X59798	523852	1.40	2.83
↑**(34) Calpain 2, (m/II) large subunit**<br>*Proteolysis and peptidolysis, cytoskeletal Remodelling, invasion*	**CAPN2**	M23254	350899	1.28	2.58
↑**(42) Macrophage migration inhibitory factor (glycosylation- inhibiting factor)**<br>*Positive regulation of angiogenesis*	**MIF**	L19686	407995	1.24	2.43
↑**(46) WD repeat domain 1 (WDR1)**<br>*Cytoskeletal remodelling, mitotic cell rounding*	**WDR1**	AF020056	128548	1.21	2.24
↑**(47) Metallothionein 1B (functional)**<br>*Regulation of Zn and Cu availability*	**MT1B**	M13485	36102	1.20	0.16
↑**(51) Interleukin 8**<br>*Positive regulation of angiogenesis*	**IL8**	M17017	624	1.18	2.05
↑**(69) A disintegrin and metalloproteinase domain 15 (metargidin)**<br>*Proteolysis and peptidolysis*	**ADAM15**	U41767	312098	1.06	1.32
↑**(88) ARP3 actin-related protein 3 homolog (yeast)**<br>*Cell motility, control of actin polymerization*	**ACTR3**	NM_005721	433512	1.00	0.96
↑**(96) Actinin, alpha 4**<br>*Cell motility, cytoskeleton dysregulation*	**ACTN4**	U48734	270291	0.96	0.79
↑**(99) Nuclear receptor subfamily 2, group F, member 2**<br>*Positive regulator of angiopoietin-1*	**NR2F2**	M64497	347991	0.95	0.54
↑**(108) Alanyl (membrane) aminopeptidase (aminopeptidase N, aminopeptidase M, microsomal aminopeptidase, CD13, p150)**<br>*Proteolysis*	**ANPEP**	M22324	1239	0.93	0.27
↑**(112) Plasminogen activator, urokinase**<br>*ECM and provisional fibrin matrix degradation*	**PLAU**	X02419/	77274	0.92	0.42
↑**(124) Actin related protein 2/3 complex, subunit 2, 34kDa**<br>*Cell motility, control of actin polymerization*	**ARPC2**	U50523	529303	0.89	0.23
↑**(130) Hepatoma-derived growth factor (high-mobility group protein 1-like)**<br>*Positive regulation of angiogenesis*	**HDGF**	L24521	506748	0.84	0.21

↓(9) **Plexin B1** *Semaphorin receptor activty and binding, cell migration*	**PLXNB1**	AJ011414	476209	-1.19	1.99
↓ (18) **Kallikrein 12** *Positive regulation of EC migration and of capillary morphogenesis*	**KLK12**	AF135025	411572	-1.08	1.39
**PROLIFERATION**					
↑(1) **Metallothionein 1A (functional)** *Regulation of Zn and Cu availability*	**MT1A**	K01383	440939	3.25	23.81
↑(8) **Connective tissue growth factor** *Positive regulation of angiogenesis, ECM synthesis*	**CTGF**	X78947	410037	1.80	6.71
↑(19) **Metallothionein 2A** *Regulation of Zn and Cu availability*	**MT2A**	V00594	418241	1.50	2.44
↑(20) **Sparc/osteonectin, cwcv and kazal-like domains proteoglycan (testican)** *Calcium ion binding: cell proliferation*	**SPOCK**	AF231124	124611	1.46	3.15
↑(21) **Metallothionein 1E (functional)** *Regulation of Zn and Cu availability*	**MT1E**	M10942	534330	1.43	2.20
↑(23) **Cyclin D1 (PRAD1: parathyroid adenomatosis 1)** *G1/S transition of mitotic cell cycle*	**CCND1**	X59798	523852	1.40	2.83
↑(47) **Metallothionein 1B (functional)** *Regulation of Zn and Cu availability*	**MT1B**	M13485	36102	1.20	0.16
↑(76) **Cysteine-rich protein 1 (intestinal)** *Zn-binding protein. Mediation of MT activity (?)*	**CRIP1**	D42123	70327	1.03	0.85
↑(112) **Plasminogen activator, urokinase** *EC proliferation, release of ECM-growth factors*	**PLAU**	X02419/	77274	0.92	0.42
↑(114) **Hypoxanthine phosphoribosyltransferase 1 (Lesch-Nyhan syndrome)** *Regulation of purine metabolism*	**HPRT1**	M31642	412707	0.91	0.54
↑(130) **Hepatoma-derived growth factor (high-mobility group protein 1-like)** *Positive regulator of EC proliferation*	**HDGF**	L24521	506748	0.84	0.21
↑(136) **Midkine (neurite growth-promoting factor 2)** *Positive regulator of EC proliferation*	**MDK**	X55110	82045	0.83	0.12
↓(6) **Kallikrein 9** *Positive regulator of EC proliferation*	**KLK9**	AF135026	448942	-1.23	2.45
↓(18) **Kallikrein 12** *Positive regulator of EC proliferation*	**KLK12**	AF135025	411572	-1.08	1.39
↓(19) **Kallikrein 11** *Positive regulator of EC proliferation*	**KLK11**	AB012917	57771	-1.08	1.39
**ADHESION**					
↑(8) **Connective tissue growth factor** *Positive regulation of angiogenesis, ECM synthesis*	**CTGF**	X78947	410037	1.80	6.71
↑(12) **Vinculin** *Cytoskeletal actin-cell membrane, cell-cell, cell-ECM interaction*	**VCL**	NM_014000	75350	1.65	5.63
↑(13) **Laminin receptor 1 (ribosomal protein SA, 67 kDa)** *Cell attachment to ECM*	**LAMR1**	U43901	374553	1.63	5.42
↑(17) **Profilin 1** *Fibronectin adhesion, focal conctat formation,*	**PFN1**	J03191	494691	1.54	4.51

cytoskeleton oragnization					
↑(20) **Sparc/osteonectin, cwcv and kazal-like domains proteoglycan (testican)** *Cell adhesion*	**SPOCK**	AF231124	124611	1.46	3.15
↑(23) **Cyclin D1 (PRAD1: parathyroid adenomatosis 1)** *Inside-out regulation of Rho-GTPases: cytoskeleton organization, adhesion*	**CCND1**	X59798	523852	1.40	2.83
↑(30) **Cofilin 1 (non-muscle)** *Actin cytoskeleton organization*	**CFL1**	X95404	180370	1.31	1.99
↑(65) **Calsyntenin 1** *Cell adhesion*	**CLSTN1**	AB020718	518451	1.07	1.41
↑(69) **A disintegrin and metalloproteinase domain 15 (metargidin)** *Cell-cell, cell-ECM adhesion*	**ADAM15**	U41767	312098	1.06	1.32
↑(117) **Zyxin** *Cell adhesion at focal contacts, actin cytoskeleton oraganization*	**ZYX**	X95735	490415	0.90	0.42
↑(122) **Integrin beta 4 binding protein** *Linking of basal lamina to the intermediate filament cytoskeleton in hemidesmosomes*	**ITGB4BP**	AF022229	534326	0.89	0.38
↓(27) **Desmoglein 2 (DSG2)** *Codherin cell adhesion molecule, homophilic cell adhesion*	**DSG2**	Z26317	412597	-1.04	1.15
↓(32) **Tenascin N** *ECM-integrin/syndecan interaction*	**TNN**	AL049689	156369	-1.01	0.43
↓(33) **Glycoprotein IX (platelet)** *Part of a cell surface receptor for von Willebrand factor*	**GP9**	X52997	1144	-1.00	0.83
↓(54) **Immunoglobulin superfamily, member 1** *Cell-cell interaction, cell-surface recognition*	**IGSF1**	AB002362	22111	-0.86	0.29
**ANGIOGENESIS INHIBITORS**					
↑ (11) **Pentaxin-related gene, rapidly induced by IL-1 beta** *Inhibitory binding of FGF-2*	**PTX3**	M31166	546280	1.66	5.19
↑ (72) **Peptidylprolyl isomerase A (cyclophilin A)-like** *Biphasic mediator of EC activation/dysfunction*	**PPIAL**	AL021395	272279	1.05	0.01
**ANGIOGENESIS UNKNOWN FUNCTIONS**					
↓ (17) **CD1B antigen, b polypeptide** *Pro-angiogenesis, induced by VEGF*	**CD1B**	M28826	1310	-1.09	1.52

M = differential expression ratio after dye-swap normalization; LOR=log odds ratio: all genes with LOR > 0 were considered significantly down-regulated (M<0) or up-regulated (M>0); in italics biological functions are reported; Each gene is univocally identifiable by a number ranging from 1 to 141 with an up-arrow meaning the up-regulation and from 1 to 58 with a down-arrow meaning the down-regulation in SSc-MVEC.
