# Supplementary material for: A model of anti-angiogenesis: differential transcriptosome profiling of microvascular endothelial cells from diffuse systemic sclerosis patients
Source: Arthritis Res Ther. 2006 Jul 19;8(4):R115. doi: 10.1186/ar2002 (PMC1779372; doi:10.1186/ar2002)
Supplement: Additional File 3 — A PDF file showing the list of differentially expressed genes involved in apoptosis, haemostasis, inflammation and immunity. This list integrates that shown in Table 2 of the text, starting from transcripts with LOR >0. [file ar2002-S3.pdf]

Additional file 3. Differentially expressed genes involved in apoptosis, haemostasis, inflammation and immunity.

Gene and biological function	Symbol	GenBank	Unigene	M	LOR
**APOPTOSIS**					
↑**(3) Tumor necrosis factor receptor superfamily, member 6b, decoy** *Secreted apoptosis inhibitor. Anti-apoptotic.*	**TNFRSF6B**	(AF104419)	434878	2.20	11.04
↑**(29) Proteoglycan 1, secretory granule** *Member of a macromolecular complex with granzymes and perforin. Pro-apoptotic.*	**PRG1**	X17042	1908	1.32	2.61
↑**(30) Cofilin 1 (non-muscle)** *Translocates to the mitochondria in the initiation phase of apoptosis. Pro-apoptotic*	**CFL1**	X95404	180370	1.31	1.99
↑**(34) Calpain 2, (m/II) large subunit** **Involved in the pathway calpain/mitochondrial** *permeability transition pore (mPTP)/Cytochrome C caspase-3. Pro-apoptotic*	**CAPN2**	M23254	350899	1.28	2.58
↑**(38) Serum/glucocorticoid regulated kinase** *Modulates the transcriptional activity of nuclear transcription factor kappa B (NF-kB). Anti-apoptotic*	**SGK**	AJ000512	296323	1.27	2.66
↑**(42) Macrophage migration inhibitory factor (glycosylation-inhibiting factor)** *Promotes phosphorylation of MEK1/2, Erk1/2,Elk-1,PI3K, Akt. Anti-apoptotic*	**MIF**	L19686	407995	1.24	2.43
↑**(44) Phosphoprotein enriched in astrocytes 15** *Mediates the Akt survival signalling. Anti-apoptotic*	**PEA15**	L37385	517216	1.23	2.36
↑**(64) Mitochondrial carrier homolog 1 (C. elegans)** *Release of cytochrome C, activation of caspase 3. Pro-apoptotic*	**MTCH1**	NM_014341	485262	1.08	1.24
↑**(106) V-akt murine thymoma viral oncogene homolog 1** *Promotes cell proliferation and survival. Anti-apoptotic*	**AKT1**	M63167	525622	0.94	0.40
↓**(20) Caspase 14, apoptosis-related cysteine protease** **Proteolytic effector of apoptosis. Pro-apoptotic**	**CASP14**	AF097874	248226	-1.07	0.96
↓**(35) Serine/threonine kinase 17a (apoptosis-inducing)** *Localized in the nucleus, induces the morphologic Changes typical of apoptosis. Pro-apoptotic*	**STK17A**	AB011420	268887	-0.98	0.71
↓**(45) BCL2-like 11 (apoptosis facilitator)** *Belongs to the family of BCL2 proteins. Anti-apoptotic*	**BCL2L11**	AF032457	469658	-0.92	0.39
↓**(48) unc-5 homolog B (C. elegans)** *Belongs to the family of netrin-1 receptors, putative tumor suppressors by control of cell death commitment. Pro-apoptotic*	**UNC5B**	AB09256	522997	-0.89	0.48
↓**(56) Sodium channel, voltage-gated, type III, beta** *Mediated a P53-dependent apoptotic pathway. Pro-apoptotic*	**SCN3B**	AB032984	4865	-0.83	0.17

**HAEMOSTASIS**					
↑(2) **Plasminogen activator, tissue** *Positive regulation of fibrinolysis*	**PLAT**	M15518	491582	2.23	11.50
↑(10) **Enolase 1, (alpha)** *Activation of plasminogen on endothelial cell surface*	**ENO1**	M14328	517145	1.67	5.79
↑(11) **Pentaxin-related gene, rapidly induced by IL-1 beta** *Increases Tissue Factor expression on endothelial cell*	**PTX3**	M31166	546280	1.66	5.19
↑(92) **Prion protein (p27-30) (Creutzfeld-Jakob disease, Gerstmann-Strausler-Scheinker syndrome, fatal familial insomnia)** *Activation of plasminogen......*	**PRNP**	U29185	472010	0.97	0.52
↑(100) **Membrane cofactor protein (CD46, trophoblast-lymphocyte cross-reactive antigen)** *Maintains the haemostatic balance of endothelial cell*	**MCP**	X59405	510402	0.95	0.74
↑(110) **Thrombomodulin** *Increases the anti-coagulant properties of the endothelial cell*	**THBD**	J02973	2030	0.93	0.62
↑(112) **Plasminogen activator, urokinase** *Positive regulation of fibrinolysis*	**PLAU**	X02419/	77274	0.92	0.42
↓(13) **Prostaglandin D2 synthase 21kDa (brain)** *Inhibitor of platelet aggregation*	**PTGDS**	NM_000954	446429	-1.14	1.84
↓(33) **Glycoprotein IX (platelet)** *Cell surface receptor for von Willebrand factor, possibly mediating platelet adhesion*	**GP9**	X52997	1144	-1.00	0.83
↓(50) **Protease, serine, 1 (trypsin 1)** *Negative regulation of blood coagulation*	**PRSS1**	M22612	511525	-0.89	0.39
**INFLAMMATION AND IMMUNITY**					
↑(11) **Pentaxin-related gene, rapidly induced by IL-1 beta** *Produced during sepsis. Complement activation and generation of inflammation mediators*	**PTX3**	M31166	546280	1.66	5.19
↑(36) **Interferon induced transmembrane protein 2 (1-8D)** *Produced in response to type I and type II interferons*	**IFITM2**	X57351	174195	1.27	2.12
↑(40) **Beta-2-microglobulin** *Component of MHC class I complex. Present in peri-vascular amyloid deposits*	**B2M**	AB021288	534255	1.26	2.56
↑(56) **Interleukin enhancer binding factor 2, 45kDa** *Transcription factor required for expression of interleukin 2 (IL-2) gene*	**ILF2**	U10323	75117	1.15	1.89
↓(3) **Lipopolysaccharide binding protein** *Upon binding with endotoxins it interacts with Toll-like receptors, thus generating inflammation mediators*	**LBP**	AF013512	154078	-1.77	6.68
↓(25) **Fc fragment of IgE, high affinity I, receptor for; alpha polypeptide** *IgE binding in allergic disease*	**FCER1A**	X06948	897	-1.04	0.84
↓ (31) **Azurocidin 1 (cationic antimicrobial protein 37)**	**AZU1**	M96326	72885	-1.02	1.11

Favors cell adhesion					
↓(58) **Fc fragment of IgG, low affinity IIa, receptor for (CD32)** *Phagocytosis of immune complexes and modulation of antibody production by B lymphocytes*	**FCGR2A**	M31932	352642	-0.82	0.12

M = differential expression ratio after dye-swap normalization; LOR=log odds ratio: all genes with LOR > 0 were considered significantly down-regulated (M<0) or up-regulated (M>0); in italics biological functions are reported; Each gene is univocally identifiable by a number ranging from 1 to 141 with an up-arrow meaning the up-regulation and from 1 to 58 with a down-arrow meaning the down-regulation in SSc-MVEC.
