# Supplementary material for: A model of anti-angiogenesis: differential transcriptosome profiling of microvascular endothelial cells from diffuse systemic sclerosis patients
Source: Arthritis Res Ther. 2006 Jul 19;8(4):R115. doi: 10.1186/ar2002 (PMC1779372; doi:10.1186/ar2002)
Supplement: Additional File 4 — A PDF file showing the list of differentially expressed genes involved in cellular stress and ubiquitination. This list integrates that shown in Table 2 of the text, starting from transcripts with LOR >0. [file ar2002-S4.pdf]

Additional file 4. Differentially expressed genes involved in cellular stress and ubiquitination.

Gene and biological function	Symbol	GenBank	Unigene	M	LOR
↑(18) **ATX1 antioxidant protein 1 homolog (yeast)**<br>*Copper chaperone ,produced in response to oxidative stress*	**ATOX1**	BT009786	125213	1.51	4.51
↑(28) **Ubiquitin carboxyl-terminal esterase L1 (ubiquitin thiolesterase)**<br>*Hydrolyzes C-terminal adducts of ubiquitin to generate ubiquitin monomer*	**UCHL1**	X04741	518731	1.33	2.99
↑(35) **APC11 anaphase promoting complex subunit 11 homolog (yeast)**<br>*Contributes to ubiquitin-protein ligase activity*	**ANAPC11**	AF151048	534456	1.28	2.76
↑(41) **Ferritin, light polypeptide**<br>*Regulates Fe homeostasis. Produced upon oxidative stress*	**FTL**	NM_000146	433670	1.25	2.35
↑(49) **Peripheral myelin protein 22 (PMP22)**<br>*Also called gas3, it is produced by endothelial cells under shear stress*	**PMP22**	D11428	372031	1.19	2.13
↑(57) **Solute carrier family 38, member 2**<br>*Aminoacid transporter. Answer to osmotic stress*	**SLC38A2**	AK001700	221847	1.14	1.28
↑(58) **Proteasome (prosome, macropain) 26S subunit, ATPase, 1**<br>*Structural member of the proteasoma, wich degrades ubiquitinated proteins*	**PSMC1**	L02426	356654	1.12	1.70
↑(59) **FK506 binding protein 4, 59kDa**<br>*Aminoacid transporter. Answer to osmotic stress. Protein folding and trafficking*	**FKBP4**	M88279	524183	1.12	1.71
↑(78) **Proteasome (prosome, macropain) 26S subunit, non-ATPase, 13 (PSMD13)**<br>*Structural member of the proteasoma, wich degrades ubiquitinated proteins*	**PSMD13**	NM_002817	134688	1.03	1.13
↑(79) **Peroxiredoxin 1**<br>*Protective anti-oxidant enzyme, produced in response to oxidative stress.*	**PRDX1**	X67951	180909	1.02	1.03
↑(97) **COP9 constitutive photomorphogenic homolog subunit 6 (Arabidopsis)**<br>*One of the eight subunits of the COP9 signalosome, involved in multiple signalling pathways. Interacts with cullin-family proteins thus regulating ubiquitin/protein ligase complexes*	**COPS6**	U70735	15591	0.96	0.26
↑(109) **Ubiquitin-conjugating enzyme E2 variant 1**<br>*Ubiquitin-coniogating enzyme activity*	**UBE2V1**	AL110132	420529	0.93	0.46
↑(113) **Neural precursor cell expressed, developmentally down-regulated 8**<br>*COP9 signalosome cleaves this protein from CUL1 subunit of ubiquitin ligases.*	**NEDD8**	D23662	531064	0.91	0.55
↑(119) **Peroxisome biogenesis factor 10**<br>*Involved in generating peroxisomes, which act as a defence against oxidative stress. Involved in ubiquitin cycle*	**PEX10**	AB013818	546273	0.89	0.04
↑(123) **Ubiquitin C**<br>*Ubiquitination*	**UBC**	M26880	520348	0.89	0.004
↑(128) **Valosin-containing protein**	**VCP**	NM_007126	529782	0.87	0.17

ATP-binding protein involved in vescicle transport, proteasome function and peroxisome assembly.Regulates ubiquitin-dependent protein degradation					
↑(131) **Cullin 2** *Regulation of the ubiquitin cycle, in cooperation with COP9 signalosome*	**CUL2**	U83410	82919	0.84	0.18
↑(132) **Proteasome (prosome, macropain) subunit, beta type, 2** *Structural member of the proteasoma, which degrades ubiquitinated proteins*	**PSMB2**	D26599	471441	0.84	0.16
↑(141) **Dual specificity phosphatase 1** *Induced by oxidative/heat stress*	**DUSP1**	NM_004417	171695	0.80	0.002
↓(8) **Anaphase promoting complex subunit 10** *Mediator of ubiquitination reactions, promotion of ubiquitinated protein catabolism*	**ANAPC10**	AL080090	480876	-1.22	2.29
↓(36) **ubiquitin specific protease 21** *Removes ubiquitin from ubiquitinated proteins*	**USP21**	AL157417	8015	-0.97	0.81
↓(55) **Glutathione peroxidase 2 (gastrointestinal)** *Induced by oxidative stress*	**GPX2**	X53463	2704	-0.84	0.19

M = differential expression ratio after dye-swap normalization; LOR=log odds ratio: all genes with LOR > 0 were considered significantly down-regulated (M<0) or up-regulated (M>0); in italics biological functions are reported; Each gene is univocally identifiable by a number ranging from 1 to 141 with an up-arrow meaning the up-regulation and from 1 to 58 with a down-arrow meaning the down-regulation in SSc-MVEC.
