# Supplementary material for: A model of anti-angiogenesis: differential transcriptosome profiling of microvascular endothelial cells from diffuse systemic sclerosis patients
Source: Arthritis Res Ther. 2006 Jul 19;8(4):R115. doi: 10.1186/ar2002 (PMC1779372; doi:10.1186/ar2002)
Supplement: Additional File 5 — A PDF file showing the list of differentially expressed genes involved in stimulus transduction, DNA/RNA organization, and transcription. This list integrates that shown in Table 2 of the text, starting from transcripts with LOR >0. [file ar2002-S5.pdf]

Additional file 5. Differentially expressed genes involved in stimulus transduction, DNA/RNA organization, transcription.

Gene and biological function	Symbol	GenBank	Unigene	M	LOR
**STIMULUS TRANSDUCTION**					
↑(24) **Gap junction protein, alpha 1, 43kDa (connexin 43)** *Activates the gap junction molecular complex. Positive regulation of I-kB kinase/NF-kB cascade*	**GJA1**	NM_000165	74471	1.39	3.55
↑(26) **Hippocalcin-like 1** *Calcium-dependent regulation of cell-signaling*	**HPCAL1**	AK000596	467696	1.36	3.36
↑(30) **Cofilin 1 (non-muscle)** *Rho phosphorylates LIM kinases, which phosphorylate cofilin, thus blocking endothelial migratory signals*	**CFL1**	X95404	180370	1.31	1.99
↑(33) **Ras-related C3 botulinum toxin substrate 2 (rho family, small GTP binding protein Rac2)** *Small GTPase-mediated signal transduction*	**RAC2**	Z82188	517601	1.30	2.90
↑(38) **Serum/glucocorticoid regulated kinase** *Protein serine/threonine kinase activity*	**SGK**	AJ000512	296323	1.27	1.27
↑(54) **Ras-GTPase-activating protein SH3-domain-binding protein** *Ras protein signal transduction*	**G3BP**	U32519	3353	1.16	0.25
↑(55) **Interleukin 6 signal transducer (gp130, oncostatin M receptor)** *Signal transducer which is part of many cytokine-receptor complex*	**IL6ST**	AL049265	532082	1.15	1.83
↑(61) **Rho GDP dissociation inhibitor (GDI) beta** *Inhibits dissociation of GDP from Rho, thus blocking Rho-dependent signal transductions*	**ARHGDIB**	L20688	504877	1.10	1.09
↑(70) **Guanine nucleotide binding protein (G protein), gamma 10** *G-protein-coupled receptor protein signalling pathway*	**GNG10**	U31383	534196	1.05	1.30
↑(71) **Peroxiredoxin 4 (PRDX4)** *Regulatory role in activation of NF-kB: depletion of NFkB activity*	**PRDX4**	U25182	83383	1.05	1.28
↑(83) **Ras homolog gene family, member A** *GTP binding, GTPase activity, Rho protein signal transduction*	**RHOA**	L09159	247077	1.02	1.10
↑(85) **Dual specificity phosphatase 3 (vaccinia virus phosphatase VH1-related)** *Inactivation of MAP kinase by de-phosphorylation: Cell growth inhibition, pro-apoptotic*	**DUSP3**	AL049417	181046	1.01	0.22
↑(93) **ADP-ribosylation factor 4** *GTP binding, GTPase activity. Small GTPase-mediated signal transduction*	**ARF4**	NM_001660	148330	0.97	0.51
↑(95) **Guanine nucleotide binding protein (G protein), beta polypeptide 1** *GTP binding, GTPase activity. Small GTPase-mediated signal transduction*	**GNB1**	X04526	430425	0.96	0.68
↑(98) **Tyrosine 3-monooxygenase/tryptophan 5-**	**YWHAE**	U54778	513851	0.95	0.71

**monooxygenase activation protein, epsilon polypeptide**<br>*Regulates signal transduction by binding to phosphoserine-containing proteins*					
↑(101) **Protein phosphatase 2 (formerly 2A), catalytic subunit, alpha isoform**<br>*Inactivation of MAP kinase by de-phosphorylation*<br>*Cell growth inhibition, pro-apoptotic*	**PPP2CA**	M60483	483408	0.95	0.45
↑(102) **Histidine triad nucleotide binding protein 1**<br>*PKC-interacting protein: inhibition of PKC*	**HINT1**	U51004	483305	0.95	0.68
↑(106) **V-akt murine thymoma viral oncogene homolog 1**<br>*MAPK signalling pathway. Toll-like receptor signalling pathway. Jak-STAT signalling pathway*	**AKT1**	M63167	525622	0.94	0.40
↑(115) **MAP kinase interacting serine/threonine kinase 2**<br>*Directly phosphorylated by ERK and MAPK. Regulation of protein synthesis by phosphorylation*<br>*of eukaryotic translation initiation factor 4E*	**MKNK2**/GPRK7	AL137615	515032	0.90	0.47
↑(116) **ADP-ribosylation factor 1**<br>*GTP binding, GTPase activity. Small GTP-ase mediated signal transduction*	**ARF1**	AK000620	286221	0.90	0.44
↑(117) **Zyxin**<br>*Messenger in the signal transduction pathways that mediates adhesion-stimulated changes in gene expression*	**ZYX**	X95735	490415	0.90	0.42
↑(129) **Parkinson disease (autosomal recessive, early onset) 7**<br>*Involved in Ras protein signal transduction*	**PARK7**	AF021819	419640	0.85	0.23
↑(135) **Exostoses (multiple) 1**<br>*Signal transduction involved in the negative regulation of cell cycle*	**EXT1**	NM_000127	492618	0.83	0.11
↑(141) **Dual specificity phosphatase 1**<br>*Inactivates target kinases by de-phosphorylation of both phosphoserine/threonine and phosphotyrosine residues.*<br>*Inactivation of MAPK/ ERK, SAPK/JNK, p38*	**DUSP1**	NM_004417	171695	0.80	0.002
↓(9) **Plexin B1**<br>*Binding of semaphorin 4D to PLXNB1 stimulates tyrosine kinase activity of MET, by acting as a GTPase-activating protein for a member of the RAS superfamily*	**PLXNB1**	AJ011414	476209	-1.19	1.99
↓(23) **Mitogen-activated protein kinase kinase kinase kinase 1**<br>*Specific mammalian STE20-like protein serine/ threonine kinase, involved in many cellular signalling cascades*	**MAP4K1**	U66464	95424	-1.05	1.20
↓(37) **5-hydroxytryptamine (serotonin) receptor 1F**<br>**Member of the G-protein-coupled receptor** *super-family. Coupled with activation of Erk/ MAPK pathways*	**HTR1F**	L05597	248136	-0.97	0.83
↓(47) **RAB5B, member RAS oncogene family**<br>*Small, monomeric GTPase, belonging to the RAS Super-family*	**RAB5B**	X54871	77690	-0.90	0.37

↓(50) **HIV-1 Rev binding protein-like**<br>*Member of a family of signal transducers with Extensive networking abilities, including Eps15-homology domain-mediate binding with SH3-containing proteins*	**HRBL**	AF053356	521083	-0.88	0.35
↓(51) **Ropporin, rhophilin associated protein 1**<br>*Protein shown to interact with Rho signalling pathway through rhophilin*	**ROPN1**	AL133624	458304	-0.87	0.35
**DNA/RNA ORGANIZATION**					
↑(5) **Shwachman-Bodian-Diamond syndrome**<br>*Rna metabolism*	**SBDS**/CGI-97	AK001779	110445	2.14	9.24
↑(10) **Enolase 1, (alpha)**<br>*Regulation of transcription*	**ENO1**	M14328	517145	1.67	5.79
↑(25) **H3 histone, family 3B (H3.3B)**<br>*Genome stabilization. Nucleosome assembly*	**H3F3B**	Z48950	180877	1.37	3.41
↑(86) **Pituitary tumor-transforming 1**<br>*Control of DNA replication and chromosome cycle and regulation of chromosome segregation*	**PTTG1**	AJ223953	350966	1.01	0.66
↑(90) **Polymerase (RNA) II (DNA directed) polypeptide L, 7. 6kDa**<br>*Regulation of transcription*	**POLR2L**	U37690	441072	0.98	0.53
↑(94) **Thioredoxin-like 4A**<br>*Component of the spliceosome complex*	**TXNL4A**	AF023612	465498	0.96	0.73
↑(103) **Suppressor of Ty 16 homolog (S. cerevisiae)**<br>*Nucleosome dys-assembly*	**SUPT16H**	NM_007192	213724	0.94	0.62
↑(111) **Heterogeneous nuclear ribonucleoprotein K**<br>*DNA and RNA binding, chromatin remodelling, transcription and splicing*	**HNRPK**	X72727	522257	0.92	0.31
↑(118) **Aurora-A kinase interacting protein**<br>*Inhibitor of Aurora-A kinases, that regulate structure and function of centrosomes and spindle*	**AKIP**	AK000615	472535	0.89	0.38
↑(120) **Structure specific recognition protein 1**<br>*Nucleosome dys-assembly and chromatin transcription elongation factor*	**SSRP1**	M86737	523680	0.89	0.36
↑(121) **RNA polymerase II associated protein 1**<br>*Function unknown*	**RPAP1**	AB037824	371045	0.89	0.36
↑(137) **5'-nucleotidase, cytosolic III**<br>*Regulation of nucleotide catabolism*	**NT5C3**	AF151067	487933	0.83	0.13
↓ (7) **TruB pseudouridine (psi) synthase homolog 2 (E.coli)**<br>*RNA modifications, control of telomerase activity*	**TRUB2**	AF131848	98541	-1.23	2.38
↓(14) **Arginine vasopressin-induced 1 (AVPI1)**<br>*Positive regulator of cell cycle*	**AVPI1**	AF131791	23918	-1.12	1.73
↓(21) **Chromosome 1 open reading frame 34**<br>*Function unknown*	**C1orf34**	AK000220	112949	-1.05	1.28
↓(26) **Myogenic factor 5**<br>*Regulation of transcription from RNA polymerase II promoter*	**MYF5**	X14894	178023	-1.04	0.25
↓(38) **Trinucleotide repeat containing 4**<br>*Regulation of alternative splicing*	**TNRC4**	U80759	26047	-0.96	0.71
↓(42) **DEAD (Asp-Glu-Ala-Asp) box polypeptide 41**<br>*Putative RNA elicase, involved in RNA processing and metabolism*	**DDX41**	AK001255	484288	-0.94	0.58

TRANSCRIPTION					
↑(45) **Basic transcription factor 3, like 3** *Transcription factor*	**BTF3L3**	M90356	181967/447132	1.21	2.14
↑(56) **Interleukin enhancer binding factor 2, 45kDa** *Transcription factor required for expression of Interleukin2 (IL-2) gene*	**ILF2**	U10323	75117	1.15	1.89
↑(84) **High mobility group AT-hook 1** *Regulator of inducible genes transcription*	**HMGA1**	L17131	518805	1.01	0.11
↑(125) **Basic transcription factor 3, like 1** **Transcription factor**	**BTF3L1**	M90354	181965	0.89	0.36
↓(1) **GATA binding protein 6** *Transcription factor, control of vWF expression*	**GATA6**	X95701	514746	-2.19	8.77
↓ (2) **SRY (sex determining region Y)-box 9 (campomelic dysplasia, autosomal sex-reversal)** *Transcription factor (unknown function in endothelial cell)*	**SOX9**	NM_000346	2316	-1.99	7.44
↓ (10) **Runt-related transcription factor 2** *Member of the Ig-loop family of transcription factors, regulates endothelial cell migration and invasion*	**RUNX2**	L40992	122116	-1.16	1.48
↓ (12) **Nuclear factor of activated T-cells 5, tonicity-responsive** *Transcription factor, important in regulation of gene expression during osmotic stress*	**NFAT5**	AF089824	371987	-1.14	1.51
↓(15) **SRY (sex determining region Y)-box 5 pseudogene** *Transcription factor (unknown function in endothelial cell)*	**SOX5P**	AF032454	248220	-1.12	1.45
↓ (16) **Forkhead box A2** *Transcription factor*	**FOXA2**	AB028021	155651	-1.10	1.59
↓(39) **Sine oculis homeobox homolog 4 (Drosophila)** *Transcription factor which binds to AU- rich elements*	**SIX4**	NM_017420	279004	-0.95	0.43
↓(44) **Nuclear factor of kappa light polypeptide gene enhancer in B-cells 2 (p49/p100)** *Transcription factor, activated by a wide variety of stimuli. Complete and persistent inhibition of NF-kB has been linked directly to apoptosis,delayed cell growth and anti-angiogenesis*	**NFKB2**	S76638	73090	-0.93	0.06

M = differential expression ratio after dye-swap normalization; LOR=log odds ratio: all genes with LOR > 0 were considered significantly down-regulated (M<0) or up-regulated (M>0); in italics biological functions are reported; Each gene is univocally identifiable by a number ranging from 1 to 141 with an up-arrow meaning the up-regulation and from 1 to 58 with a down-arrow meaning the down-regulation in SSc-MVEC.
