# Supplementary material for: A model of anti-angiogenesis: differential transcriptosome profiling of microvascular endothelial cells from diffuse systemic sclerosis patients
Source: Arthritis Res Ther. 2006 Jul 19;8(4):R115. doi: 10.1186/ar2002 (PMC1779372; doi:10.1186/ar2002)
Supplement: Additional File 6 — A PDF file showing the list of differentially expressed genes involved in regulation of protein synthesis and mitochondrial functions. Genes involved in protein synthesis were identified as structural components of the ribosome or as functional regulators of protein synthesis. All of them were up-regulated in SSc-MVECs, as well as transcripts regulating mitochondrial functions. [file ar2002-S6.pdf]

Additional file 6. Differentially expressed genes involved in regulation of protein synthesis and mitochondrial functions.

Gene and biological function	Symbol	GenBank	Unigene	M	LOR
**PROTEIN SYNTHESIS**					
**↑(7) Eukaryotic translation elongation factor 1 beta 2** *Regulates transfer of aminoacylated tRNA to ribosome*	**EEF1B2**	X60656	421608	1.84	7.48
**↑(9) Ribosomal protein L38** *Structural constituent of ribosome*	**RPL38**	Z26876	380953	1.70	5.20
**↑(15) Ribosomal protein L12** *Structural constituent of ribosome*	**RPL12**	AF037643	408054	1.57	4.27
**↑(22) Ribosomal protein L9** *Structural constituent of ribosome*	**RPL9**	U09953	412370	1.41	2.76
**↑(27) Ribosomal protein S20** *Structural constituent of ribosome*	**RPS20**	L06498	8102	1.34	2.33
**↑(43) Ribosomal protein L23a** *Structural constituent of ribosome*	**RPL23A**	U37230	184776	1.23	2.35
**↑(48) Ribosomal protein L10** *Structural constituent of ribosome*	**RPL10**	M64241	401929	1.20	0.82
**↑(50) Ribosomal protein L7** *Structural constituent of ribosome*	**RPL7**	X57958	421257	1.18	1.89
**↑(67) Ribosomal protein S5** *Structural constituent of ribosome*	**RPS5**	U14970	378103	1.07	0.49
**↑(74) Ribosomal protein S10 (RPS10)** *Structural constituent of ribosome*	**RPS10**	U14972	406620	1.03	0.86
**↑(82) Ribosomal protein L10a** *Structural constituent of ribosome*	**RPL10A**	NM_007104	14834	1.02	0.90
**↑(107) Ribosomal protein L14** *Structural constituent of ribosome*	**RPL14**	D87735	446522	0.94	0.63
**↑(122) Integrin beta 4 binding protein** *Translation initiation factor*	**ITGB4BP**	AF022229	534326	0.89	0.38
**↑(126) Nucleolar protein family A, member 2 (H/ACA small nucleolar RNPs)** *Ribosome biogenesis and assembly*	**NOLA2**	AK000486	27222	0.89	0.16
**↑(134) Eukaryotic translation initiation factor 4 gamma, 1** *Recognition of mRNA cap, recruitment of mRNA to the ribosome*	**EIF4G1**	AF104913	433750	0.84	0.17
**MITOCHONDRIAL FUNCTIONS**					
**↑ 52 NADH dehydrogenase (ubiquinone) Fe-S protein 5, 15kDa (NADH-coenzyme Q reductase)** *Oxidative phosphorylation*	**NDUFS5**	AF047434	472185	1.17	2.03
**↑ 62 NADH dehydrogenase (ubiquinone) 1 alpha subcomplex, 8, 19kDa** *Oxidative phosphorylation*	**NDUFA8**	NM_014222	495039	1.10	1.62
**↑ 63 ATP synthase, H+ transporting, mitochondrial F0 complex, subunit c (subunit 9), isoform 1** *ATPase activity, proton transport*	**ATP5G1**	X69907	80986	1.09	1.54
**↑ 73 NADH dehydrogenase (ubiquinone) 1 alpha subcomplex, 6, 14kDa** *Oxidative phosphorylation*	**NDUFA6**	AF047182	274416	1.03	1.20
**↑ 87 Ubiquinol-cytochrome c reductase hinge**	**UQCRH**	Y00764	481571	1.00	0.84

**protein**<br>*Oxidative phosphorylation*					
↑ **91 Solute carrier family 9 (sodium/hydrogen exchanger), isoform 6**<br>*Ion transport and pH regulation in mitochondrion, microsomes, endoplasmic reticulum*	**SLC9A6**	AF030409	62185	0.98	0.56
↑ **105 Solute carrier family 25 (mitochondrial carrier; phosphate carrier), member 3**<br>*Transport of phosphate into the mitocondrial matrix*	**SLC25A3**	NM_005888	290404	0.94	0.43
↑ **127 Translocase of inner mitochondrial membrane 8 homolog B (yeast)**<br>*Protein translocation through mitocondrial membrane*	**TIMM8B**	NM_012459	279915	0.87	0.28
↑ **133 cytochrome c oxidase subunit VIa polypeptide 1**<br>*Oxidative phosphorylation*	**COX6A1**	AL021546	497118	0.84	0.16
↑ **148 Succinate dehydrogenase complex, subunit D, integral membrane protein**<br>*Regulation of the activity of mitocondrial complex II*	**SDHD**	AB006202	356270	0.82	0.05

M = differential expression ratio after dye-swap normalization; LOR=log odds ratio: all genes with LOR > 0 were considered significantly down-regulated (M<0) or up-regulated (M>0); in italics biological functions are reported; Each gene is univocally identifiable by a number ranging from 1 to 141 with an up-arrow meaning the up-regulation and from 1 to 58 with a down-arrow meaning the down-regulation in SSc-MVEC.
