# Supplementary material for: A model of anti-angiogenesis: differential transcriptosome profiling of microvascular endothelial cells from diffuse systemic sclerosis patients
Source: Arthritis Res Ther. 2006 Jul 19;8(4):R115. doi: 10.1186/ar2002 (PMC1779372; doi:10.1186/ar2002)
Supplement: Additional File 7 — A PDF file showing the list of differentially expressed genes with unknown function or that cannot be included within a class. Nineteen transcripts were up-regulated and 17 down-regulated in SSc-MVECs. [file ar2002-S7.pdf]

Additional file 7. Differentially expressed genes with function unknown or that cannot be included within a class.

Gene and biological function	Symbol	GenBank	Unigene	M	LOR
**↑(5) Gamma-glutamyl hydrolase (conjugase, folylpolygammaglutamyl hydrolase)** *Lysosome exopeptidase*	**GGH**	U55206	78619	2.03	8.41
**↑(14) Hypothetical gene supported by AF131741** *Unknown function*	**LOC441052**	AF131741	535012	1.58	3.92
**↑(16) Phosphoglycerate mutase 1 (brain)** *Glycolysis*	**PGAM1**	J04173	447492	1.55	4.33
**↑(31) Zinc finger CCCH type domain containing 1** *Unknown function*	**ZC3HDC1**	AL13725	12646	1.30	2.43
**↑(32) Microsomal glutathione S-transferase 3** *Glutathione transferase activity*	**MGST3**	AF026977	191734	1.30	2.93
**↑(37) Hematological and neurological expressed 1** *Haematopoietic cells and neurological development*	**HN1**	NM_016185	532803	1.27	2.59
**↑(40) Chromosome 13 open reading frame 12 (C13orf12)** *Unknown function*	**C13orf12**	NM_015932	268742	1.25	2.49
**↑(53) Adaptor-related protein complex 2, sigma 1 subunit** *Clathrin cage assembly and endocytosis regulator*	**AP2S1**	X97074	119591	1.17	2.00
**↑ (60) ATPase, H+ transporting, lysosomal 56/58kDa, V1 subunit B, isoform 2** *ATPase-dependent proton transport*	**ATP6V1B2**	L35249	295917	1.11	1.70
**↑ (66) DKFZp564J157 protein** *Unknown function*	**DKFZp564J157**	AL122042	426359	1.07	1.40
**↑(68) Transmembrane protein 14C** *Unknown function*	**TMEM14C**	AF151028	519557	1.06	1.38
**↑ (75) EGF-containing fibulin-like extracellular matrix protein 1 (EFEMP1)** *Repressed during capillary morphogenesis*	**EFEMP1**	U03877	76224	1.03	1.17
**↑(77) Peroxisomal long-chain acyl-coA thioesterase** *Acyl-CoA methabolism*	**ZAP128**	AK001939	446685	1.03	1.14
**↑(80) Hypothetical protein LOC348262** *Unknown function*	**LOC348262**	AK000852	514632	1.02	1.14
**↑(81) ATP citrate lyase** *Synthesis of cytosolic acetyl-CoA*	**ACLY**	X64330	387567	1.02	1.15
**↑(89) Esterase D/formylglutathione hydrolase** *Unknown function*	**ESD**	AF112219	432491	0.98	0.78
**↑ (104) Major vault protein** *Drug resistance*	**MVP**	X79882	513488	0.94	0.48
**↑ (139) Hypothetical protein LOC116068** *Cell wall catabolism*	**LOC116068**	AL137315	136235	0,82	0,07
**↑(140) Aminopeptidase puromycin sensitive** *Zinc metallopeptidase possibly involved in proteolytic events regulating the cell cycle*	**NPEPPS**	AJ132583	443837	0.81	0.03
**↓ (4) Apolipoprotein B mRNA editing enzyme, catalytic polypeptide-like 3B apolipoprotein B mRNA editing enzyme, catalytic polypeptide-like 3A** *Control of cell cycle and cell growth*	**APOBEC3B APOBEC3A**	AL022318	226307	-1.25	2.36
**↓(5) Sperm associated antigen 6**	**SPAG6**	AF079363	527698	-1.23	2.47

Control of sperm flagellar motility and structural integrity					
**↓(11) Folate receptor 2 (fetal)** *Member of the folate receptor (FOLR) family*	**FOLR2**	AF000380	433159	-1.15	1.84
**↓(22) Apolipoprotein A-I** *High-density lipoprotein binding and lipoprotein methabolism*	**APOA1**	X02162	93194	-1.05	0.93
**↓(24) Hypothetical gene supported by AL133018** *Unknown function*	**LOC401018**	AL133018	534786	-1.05	0.85
**↓(28) Glycine N-methyltransferase** *Regulation of methionine methabolism*	**GNMT**	X62250	144914	-1.03	1.08
**↓(29) Myotubularin related protein 8** *Hydrolytic enzyme involved in phospholipids and protein amino acid dephosphorylation*	**MTMR8**	AF073482	442892	-1.02	0.82
**↓(30) PTD013 protein/ RWD domain containing 1** *Unknown function*	**RWDD1**	NM_016104	532164	-1.02	1.01
**↓(34) Hypothetical protein FLJ10769** *Unknown function*	**FLJ10769**	AK001631	408324	-1.00	0.99
**↓(40) Leucine-rich repeats and calponin homology (CH) domain containing 4** *Putative receptor with unknown function*	**LRCH4**	AF053356	125742	-0.94	0.72
**↓(41) Cytochrome P450, family 2, subfamily C, polypeptide 18** *Protein localized to the endoplasmic reticulum. Specific substrate not yet determined*	**CYP2C18**	M61853	511872	-0.94	0.16
**↓(43) Hypothetical protein MGC11332** *Tetracycline transport*	**MGC11332**	U79290	98798	-0.93	0.64
**↓(46) Hypothetical protein FLJ11142** *Unknown function*	**FLJ11142**	AL133606	98704	-0.91	0.51
**↓(49) Putative lymphocyte G0/G1 switch gene** *Unknown function*	**G0S2**	M69199	432132	-0.89	0.05
**↓(52) Vitelliform macular dystrophy (Best disease, bestrophin)** *Ion transport*	**VMD2**	AF057170	132319	-0.87	0.33
**↓(57) Spectrin repeat containing, nuclear envelope 1** *Nuclear and Golgi complex organization*	**SYNE1**	AL049548	12967	-0.83	0.08
**↓(59) Hypothetical protein MGC27085** *Unknown function*	**MGC27085**	BC028610	120277	-0.82	0.06

M = differential expression ratio after dye-swap normalization; LOR=log odds ratio: all genes with LOR > 0 were considered significantly down-regulated (M<0) or up-regulated (M>0); in italics biological functions are reported; Each gene is univocally identifiable by a number ranging from 1 to 141 with an up-arrow meaning the up-regulation and from 1 to 58 with a down-arrow meaning the down-regulation in SSc-MVEC.
